# Unveiling the importance of nonshortest paths in quantum networks

**DOI:** 10.1126/sciadv.adt2404

**Published:** 2025-02-26

**Authors:** Xinqi Hu, Gaogao Dong, Kim Christensen, Hanlin Sun, Jingfang Fan, Zihao Tian, Jianxi Gao, Shlomo Havlin, Renaud Lambiotte, Xiangyi Meng

**Affiliations:** ^1^ School of Mathematical Sciences, Jiangsu University, Zhenjiang, Jiangsu 212013, China.; ^2^ Key Laboratory for NSLSCS, Ministry of Education, School of Mathematical Sciences, Nanjing Normal University, Nanjing 210023, China.; ^3^ Blackett Laboratory and Centre for Complexity Science, Imperial College London, London SW7 2AZ, UK.; ^4^ Nordita, KTH Royal Institute of Technology and Stockholm University, Hannes Alfvéns väg 12, SE-106 91 Stockholm, Sweden.; ^5^ School of Systems Science and Institute of Nonequilibrium Systems, Beijing Normal University, Beijing 100875, China.; ^6^ School of Management and Engineering, Nanjing University, Nanjing 210093, China.; ^7^ Network Science and Technology Center, Rensselaer Polytechnic Institute, Troy, NY 12180, USA.; ^8^ Department of Computer Science, Rensselaer Polytechnic Institute, Troy, NY 12180, USA.; ^9^ Department of Physics, Bar-Ilan University, Ramat Gan 52900, Israel.; ^10^ Mathematical Institute, University of Oxford, Oxford OX2 6GG, UK.; ^11^ Turing Institute, London NW1 2DB, UK.; ^12^ Department of Physics, Applied Physics, and Astronomy, Rensselaer Polytechnic Institute, Troy, NY 12180, USA.

## Abstract

Quantum networks (QNs) exhibit stronger connectivity than predicted by classical percolation, yet the origin of this phenomenon remains unexplored. We apply a statistical physics model—concurrence percolation—to uncover the origin of stronger connectivity on hierarchical scale-free networks, the (*U*, *V*) flowers. These networks allow full analytical control over path connectivity through two adjustable path-length parameters, ≤*V*. This precise control enables us to determine critical exponents well beyond current simulation limits, revealing that classical and concurrence percolations, while both satisfying the hyperscaling relation, fall into distinct universality classes. This distinction arises from how they “superpose” parallel, nonshortest path contributions into overall connectivity. Concurrence percolation, unlike its classical counterpart, is sensitive to nonshortest paths and shows higher resilience to detours as these paths lengthen. This enhanced resilience is also observed in real-world hierarchical, scale-free internet networks. Our findings highlight a crucial principle for QN design: When nonshortest paths are abundant, they notably enhance QN connectivity beyond what is achievable with classical percolation.

## Teaser

Contrary to classical expectation in network theory, quantum networks can be boosted by exploiting nonshortest path connectivity.

## INTRODUCTION

**Fig. 1.**
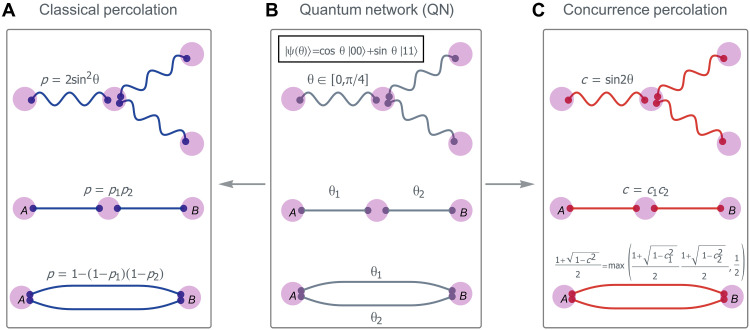
Percolation mappings of a quantum network (QN). (**A** to **C**) In a QN, a node comprises several qubits, each entangled with those in other nodes, and a link symbolizes a partially entangled bipartite state ∣ψ(θ)〉 shared between two nodes. Entanglement transmission across QN can be understood based on two distinct statistical physics mappings: (A) classical percolation (blue) versus (C) concurrence quantum percolation (red). Each mapping is based on its own set of series and parallel path connectivity rules which dictate the superposition of multiple path connectivities (*55*) to determine the total percolation connectivity between two distant nodes, say, Alice (node *A*) and Bob (node *B*).

The emerging prospect of quantum internet (*1*) is driving the field of quantum computation and communication to explore larger and more complex scales, entering the realms of statistical physics (*2*) and network science (*3*). This promising potential of quantum communication relies on the ability of transmitting quantum resources—most notably, entanglement (*4*)—across vast distances. Entanglement is fundamental in various quantum information tasks, including distributed quantum computation (*5*), where qubits act as entangled remote proxies via quantum teleportation (*6*), and unbreakable quantum secret sharing (*7*), which leverages the no-cloning principle (*8*). However, in practical scenarios, real-world entanglement is often imperfect and susceptible to noise, which poses substantial challenges for long-distance transmission (*9*). To investigate how imperfect entanglement behaves across complex network topologies, a quantum network (QN) model (*10*) has been introduced. In the QN model, each link represents an identical, partially entangled state ∣ψ(θ)〉=cosθ∣00〉+sinθ∣11〉, shared between two qubits that are situated at the nodes associated with the link (Fig. 1). The parameter θ ∈ [0, π/4] serves as a “link weight” that quantifies the degree of entanglement. Specifically, θ = π/4 signifies a maximally entangled state, while θ = 0 indicates zero entanglement. This homogeneous, pure-state QN maps onto a classical percolation problem (*10*), where each link can use certain quantum operations as a sort of “gambling” to enhance its level of entanglement, allowing a probability $p\equiv 2{\text{sin}}^{2}\theta \in \left[0,1\right]$ of becoming perfectly entangled and 1 − *p* of losing all entanglement (*10*). Within this percolation analogy, entanglement transmission between two distant nodes—say, Alice (*A*) and Bob (*B*)—translates to the probability of having a path comprising only maximally entangled links between *A* and *B*. The presence of such a path further ensures that infinite-distance entanglement can be established between *A* and *B* (*11*), marking a successful event of entanglement transmission.

In the thermodynamic limit (i.e., when the number of nodes *N* → ∞), classical percolation exhibits a critical threshold *p*_th_ (*12*, *13*). It implies that if $\theta <{\text{sin}}^{-1}\sqrt{p_{\text{th}}/2}$, no entanglement transmission can happen between *A* and *B* when their distance tends to infinity. This is, however, not the case in the QN. Here, a more effective mapping from QN to a percolation-like statistical theory of concurrence percolation has been found (*11*). This mapping, while referred to as percolation, is based on a different framework that moves away from the conventional cluster-based concepts such as cluster-size distribution. Instead, it is grounded in Kesten’s original treatment of classical percolation, which focuses on paths (*14*). Within its path-based framework, both classical and concurrence “quantum” percolating connectivity can be understood as a “superposition” of the contributions of all possible paths connecting nodes *A* and *B*. In classical percolation, the overall probability of connection of *A* and *B* can be computed through each individual path, contingent upon the parameter $p=2{\text{sin}}^{2}\theta \in \left[0,1\right]$. The superposition can be simplified into connectivity rules, such as the series and parallel rules (Fig. 1A). Similarly, in concurrence percolation, a distinct parameter *c* denoting concurrence, a conventional measure of entanglement, can be defined via θ in the QN, c≡sin2θ∈[0,1]. The superposition of concurrence paths adheres to a distinct set of connectivity rules (Fig. 1C). Concurrence percolation also reveals a critical threshold *c*_th_, which, in terms of θ, is always lower than *p*_th_: $\left({\text{sin}}^{-1}c_{\text{th}}\right)/2\leq {\text{sin}}^{-1}\sqrt{p_{\text{th}}/2}$ (*15*), indicating that QNs have a stronger connectivity than classical percolation predicts.

A challenging and fundamental question thus arises. What is the origin of this stronger connectivity, from a statistical physics perspective? It is conceivable that concurrence percolation might simply be a variant of classical percolation (albeit under a different set of variables), thereby belonging to the same universality class. Alternatively, concurrence percolation could represent a fundamentally distinct phenomenon from classical percolation, characterized by distinct critical exponents. Previous research, unfortunately, has been unable to identify the universality class of concurrence percolation due to computational constraints (*16*). Gaining a deeper insight into the nature of this stronger concurrence connectivity could shed light on QN design from first principles, as explored through the lens of network science.

This study examines the critical phenomena of concurrence percolation in networks with hierarchical and scale-free structures (*17*, *18*), which are typical characteristics of many real-world networks, including the internet (*19*), transportation networks (*20*), and brain networks (*21*, *22*). First, we exactly determine the critical exponents for a family of hierarchical scale-free network models known as the (*U*, *V*) flowers (*23*, *24*), which are characterized by two distinct network length scales, *U* ≤ *V*. The (*U*, *V*) flowers provide a simple model for studying the influence of varying length scales through both analytical and numerical methods. Our analysis firmly shows that concurrence quantum percolation belongs to a universality class distinct from that of classical percolation.

We highlight that this separation of universality classes is rooted in how the two percolation problems respond to an increase in the longer length scale, *V*, controlling the lengths of nonshortest paths. When *V* → ∞, we find that the classical percolation critical exponents become decoupled from *V*, depending only on the shorter length scale *U*. In contrast, the concurrence percolation critical exponents depend on both *U* and *V*. This distinction extends to the behavior of critical thresholds: While both *p*_th_ and *c*_th_ tend toward unity as *V* → ∞, the concurrence threshold *c*_th_ has a slower rate of approach. This implies a higher resilience (*25*–*27*) of concurrence connectivity against increase of *V*, a phenomenon we also observe in real-world network topology. Our findings emphasize the role of nonshortest paths in QN: Despite the exponential decay of entanglement along longer paths, these paths cannot be ignored in concurrence percolation. If abundant, nonshortest paths still contribute notably to QN connectivity. In practice, this principle suggests that an effective design of QN should move beyond focusing solely on the shortest paths to strategic incorporation of longer paths as well. This view may open up opportunities to explore advanced quantum communication technologies, such as path routing (*28*) and network coding (*29*), in a synergistic manner. By highlighting the role of nonshortest paths, this work provides insight into the fundamental mechanism driving the superior performance of QNs and offers guidance for designing robust QNs.

## RESULTS

### Critical exponents and hyperscaling

**Fig. 2.**
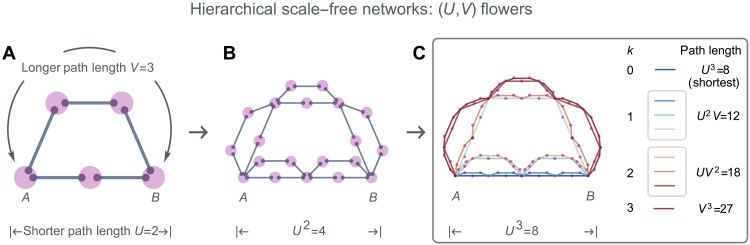
(*U*, *V*) flowers as hierarchical scale-free networks. The (*U*, *V*) flower is a self-similar network, constructed by iteratively replacing each link with a motif comprising two nodes connected by two parallel paths with lengths *U* ≤ *V*, respectively. The *n*th generation (*U*, *V*) flower can be fully decomposed into 2^*n*^ nonoverlapping paths between nodes *A* and *B*, with varying lengths $\left\{U^{n},U^{n-1}V,\ldots ,V^{n}\right\}$ and corresponding count of paths $\left\{C_{n}^{0},C_{n}^{1},\ldots ,C_{n}^{n}\right\}$, where $C_{n}^{k}=\frac{n!}{k!\left(n-k\right)!}$. The example shows *U* = 2 and *V* = 3 for (**A**) *n* = 1, (**B**) *n* = 2, and (**C**) *n* = 3. In (C), the eight nonoverlapping paths between *A* and *B* are indicated with different colors.

We begin by focusing on (*U*, *V*) flowers (*23*), a canonical example of hierarchical, scale-free networks. In a (*U*, *V*) flower, the (*n* + 1)th generation is built by replacing each existing link in the *n*th generation by a basic motif, formed by a shorter path with *U* links and a longer path with *V* links between two nodes (Fig. 2). The two scales *U* and *V* determine different network characteristics: while the shorter length scale *U* governs the unique shortest path length

$$L=U^{n}$$ (1)

between nodes *A* and *B*, which is asymptotically the same as the diameter of the network (*24*), the longer length scale *V* controls the lengths of all other nonshortest paths, thereby controlling the fractal dimension of the network

$$d=\underset{n\to \infty }{\text{lim}}\frac{\text{ln}N}{\text{ln}L}=\frac{\text{ln}\left(U+V\right)}{\text{ln}U}$$ (2)

where *N* ∼ (*U* + *V*)^*n*^ is the number of nodes in the network (*24*). We find that the (*U*, *V*) flower is not hyperbolic except in the case of *U* = 1 (section S1). Hyperbolic networks, known for their distinctive critical phenomena such as Berezinskii-Kosterlitz-Thouless–type transitions and discontinuous percolation behaviors (*30*–*36*), are beyond the scope of this study. Therefore, we exclude *U* = 1 from our analysis and only consider 1 < *U* ≤ *V*.

An interesting feature of (*U*, *V*) flowers is that they are series-parallel networks (*37*). Consequently, they allow for an analytical calculation of the sponge-crossing probability in classical percolation (*14*, *38*), *P*_SC_ [a.k.a. the spanning probability $\Pi_{\infty }\left(p;L\right)$ in boundary conformal field theories (*39*)]. For (*U*, *V*) flowers, *P*_SC_ denotes the probability of connection between the two boundary nodes *A* and *B*. Specifically, *P*_SC_ can be calculated through iteratively applying an exact renormalization group (RG) function that is constructed from series and parallel rules (Fig. 1A)

$$R\left(p\right)=\text{para}\left(\text{seri}\left(\overset{U}{\overbrace{p,p,\ldots ,p}}\right),\text{seri}\left(\overset{V}{\overbrace{p,p,\ldots ,p}}\right)\right)$$ (3)

Equation 3 allows us to derive the *n*th generation *P*_SC_ by nesting R a total of *n* times, given by $P_{\text{SC}}=\overset{n}{\overbrace{R(R(R(\ldots R}}\left(p\right))))$. The critical threshold *p*_th_ can be determined by finding the nontrivial fixed point p* that satisfies the RG fixed point equation, $R\left(p^{*}\right)=p^{*}$.

**Fig. 3.**
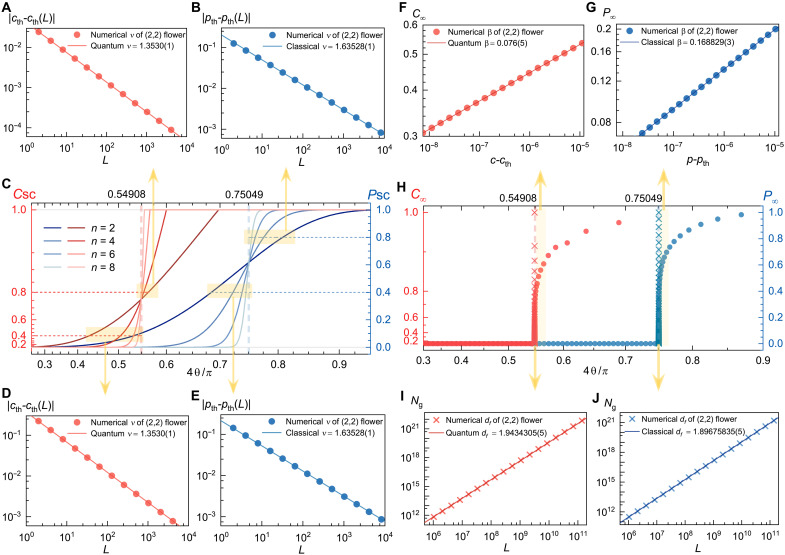
Critical phenomena on (*U*, *V*) flowers. (**A** and **B**) Scaling of critical exponent ν in quantum (red) and classical (blue) mappings above the thresholds, where *c*_th_(*L*) and *p*_th_(*L*) are solved at *C*_SC_ = 0.8 and *P*_SC_ = 0.8, respectively, and *n* = 1, 2, …, 13. (**C**) Classical and concurrence sponge-crossing percolation of generation *n* = 2,4,6,8 on (2, 2) flowers. The dashed vertical lines are at the critical thresholds. (**D** and **E**) Scaling of ν in quantum and classical mappings below the thresholds, where *c*_th_(*L*) and *p*_th_(*L*) are solved at *C*_SC_ = 0.4 and *P*_SC_ = 0.4, respectively and *n* = 1, 2, …, 13. (**F** and **G**) Scaling of classical and quantum critical exponent β. (**H**) Percolating strengths *P*_∞_ and *C*_∞_ on (2, 2) flowers for *n* = 150. (**I** and **J**) Scaling of “percolating cluster” $N_{g}={\mathrm{NP}}_{\infty }\propto L^{d_{f}}$ (classical) and $N_{g}={\mathrm{NC}}_{\infty }\propto L^{d_{f}}$ (quantum) with network diameter *L*. The cross symbols are the percolation value at the critical threshold for *n* = 20, 21, …, 37.

Similarly, the sponge-crossing concurrence *C*_SC_ and the concurrence threshold *c*_th_ (hereafter referred to as quantum results) can be calculated by replacing *P*_SC_ by *C*_SC_ in Eq. 3 and the corresponding classical series/parallel rules to their quantum counterparts (Fig. 1C). For example, in a (2, 2) flower, for concurrence percolation, we find *c*_th_ = 0.759…, or θ ≈ 0.549π/4, while for classical percolation, $p_{\text{th}}=\left(\sqrt{5}-1\right)/2\approx 0.618$ (*24*), corresponding to θ ≈ 0.750π/4 (Fig. 3C). This is an explicit example showing that the quantum threshold is lower than the classical one (*11*). Furthermore, we show that the critical threshold is unique for both classical and quantum percolation (section S2), indicative of an ordinary second-order continuous phase transition.

Near criticality, the thermal exponent ν characterizes the divergence of the correlation length as one approaches the critical threshold. We can obtain an exact expression for ν-formula> by evaluating [40]

$${\left.\frac{\partial R\left(p\right)}{\partial p}\right|}_{p=p_{\text{th}}}=U^{1/\nu }\Rightarrow \nu =\frac{\text{ln}U}{\text{ln}\left[{\left.\frac{\partial R\left(p\right)}{\partial p}\right|}_{p=p_{\text{th}}}\right]}$$ (4)

where *U* corresponds to the length scale that is associated with the network diameter (see Eq. 1). Compared to the established classical value of ν = 1.635 … (*24*), we find a quantum value of ν = 1.352 …. These exact values are numerically verified by finite-size scaling analysis: Assuming that the finite-size critical threshold *p*_th_(*L*) deviates from the true threshold *p*_th_ by $|p_{\text{th}}\left(L\right)-p_{\text{th}}|∼L^{-1/\nu }$, we find ν ≈ 1.63528(1) for classical percolation and ν ≈ 1.3530(1) for quantum percolation on both sides of the critical threshold of the (2, 2) flower (Fig. 3, A, B, D, and E).

In the traditional study of classical percolation, not only does the length scale but the cluster size distribution shows critical behaviors as well. Notably, the emergence of a unique infinite percolating cluster when *p* > *p*_th_ leads to a scaling behavior *P*_∞_ ∼ (*p* − *p*_th_)^β^ for $p\to p_{\text{th}}^{+}$, where the percolating strength *P*_∞_ = *N*_*g*_/*N* represents the ratio of the percolating cluster’s relative size *N*_*g*_ to the total network size *N*, that is, the probability that a randomly chosen node belongs to the percolating cluster (*40*). This cluster-based concept of *P*_∞_ has not been translated into the quantum counterpart, which relies on path connectivity rules and lacks a notion of “clusters.” Nevertheless, we notice that *P*_∞_ can be alternatively considered as the probability of a randomly chosen node in the bulk to reach the boundaries of the network (*24*). This consideration allows us to redefine *P*_∞_, and crucially *C*_∞_, in terms of path connectivity from a randomly chosen node (which must be averaged across all nodes) to the boundaries (which are assumed to exist). For the (*U*, *V*) flowers, the boundaries are represented by *A* and *B*. The construction is detailed in sections S3 and S4.

Using this alternative definition of *P*_∞_, we find an exponent β ≈ 0.168829(3) for classical percolation (Fig. 3G), closely matching the exact cluster-defined value, β = 0.165 … on (2, 2) flowers (*24*). The numerical results do not agree exactly with the theoretical value because computing *P*_∞_ requires higher-order path connectivity rules that extend beyond the basic series and parallel rules. We approximate these higher-order rules using only the series/parallel rules through a technique known as the star-mesh transform (*11*, *41*), which yields results that are close, but not exact (section S6). In the quantum domain, we observe β ≈ 0.076(5) (Fig. 3F), markedly distinct and smaller than its classical counterpart, suggesting an almost discontinuous transition that is comparable to explosive percolation [β ≈ 0.0555(1)] (*42*, *43*).

Another critical exponent, *d*_*f*_, defines the fractal dimension of the percolating cluster at the critical threshold, with $N_{g}∼L^{d_{f}}$. Again, lacking a cluster notion, *N*_*g*_ = *NP*_∞_ must be redefined through *P*_∞_ at the critical threshold. We find *d*_*f*_ ≈ 1.89675835(5) (Fig. 3J) in the classical scenario, very close to the cluster-defined value *d*_*f*_ = 1.899 … (*24*). In contrast, we find that the quantum scenario shows *d*_*f*_ ≈ 1.9434305(5) (Fig. 3I).

The three path-defined critical exponents still adhere to the hyperscaling relation, *d*_*f*_ = *d* − β/ν (*44*), in both classical and concurrence percolation (section S5), implying a consistent path-connectivity framework across different percolation theories. As an example, a summary of the critical exponents for the (2, 2) flower is shown in Table 1. Critical exponents of a general (*U*, *V*) flower can be obtained via the same approach (section S6). See details in Table 2 for results in the *V* → ∞ limit and section S6.

**Table 1.** Numerical critical exponents for *U* = *V* = 2. *d*, network dimension; ν, characterizing divergence of the correlation length when $p\to p_{\text{th}}^{\pm }$ ($c\to c_{\text{th}}^{\pm }$); *d*_*f*_, scaling of the percolating cluster size *N*_*g*_ = *NP*_∞_ (*NC*_∞_) with system size *N* at *p* = *p*_th_ (*c* = *c*_th_); β, characterizing symmetry breaking of the percolating strength *P*_∞_ (*C*_∞_) when $p\to p_{\text{th}}^{+}$ ($c\to c_{\text{th}}^{+}$); *d*_−_*d*_*f*_ − β/ν, equal to zero when the hyperscaling relation is satisfied. Numbers in parentheses represent standard errors.

	Classical percolation	Concurrence quantum percolation
*d*	2	2
ν	1.63528(1)	1.3530(1)
*d*_*f*_	1.89675835(5)	1.9434305(5)
β	0.168829(3)	0.076(5)
*d* − *d*_*f*_ − β/ν	3 × 10^−9^ (2 × 10^−6^)	4 × 10^−4^ (4 × 10^−3^)

**Table 2.** Critical exponents in the *V* → ∞ limit. Analytical and numerical asymptotic analyses are provided in section S6.

	Classical [∼*f*(*U*) + *O*(*V*^−1^)]	Quantum [∼*g*(*U*, *V*)]
ν	$\frac{\text{ln}U}{\text{ln}\left[1+\left(U-1\right)\text{ln}\frac{U}{U-1}\right]}+O\left(V^{-1}\right)$	$\frac{\text{ln}U}{\text{ln}\text{ln}V}+O\left[\frac{\text{lnlnln}V}{{\left(\text{lnln}V\right)}^{2}}\right]$
*d* − *d*_*f*_	$\frac{\text{ln}U-\text{ln}\left[\left(1-U\right)+2/\left(\text{ln}\frac{U}{U-1}\right)\right]}{\text{ln}U}+O\left(V^{-1}\right)$	$\frac{\text{ln}\text{ln}V}{\text{ln}U}+O\left(\frac{\text{ln}\text{ln}V}{\text{ln}V}\right)$
β	$\frac{\text{ln}U-\text{ln}\left[\left(1-U\right)+2/\left(\text{ln}\frac{U}{U-1}\right)\right]}{\text{ln}\left[1+\left(U-1\right)\text{ln}\frac{U}{U-1}\right]}+O\left(V^{-1}\right)$	$1+O\left(\frac{\text{ln}\text{ln}\text{ln}V}{\text{ln}\text{ln}V}\right)$

Note that all length scales are established in the chemical-distance (“time”) space (*23*), thus differing from traditional Euclidean length scales ξ by an additional exponent *z*, related by *L* ∼ ξ^*z*^. In other words, our values of *d*, *d*_*f*_, and ν correspond to *d*/*z*, *d*_*f*_/*z*, and zν in the Euclidean scale (*45*).

### Asymptotic dependence on the longer length scale

To investigate the impact of the longer length scale *V* and the shorter length scale *U* on the two percolation theories, we derive the asymptotic behaviors of both critical thresholds *p*_th_ and *c*_th_ in the *V* → ∞ limit using constant *U* > 1 and observe the simulation values of percolation critical exponent for finite *U* when *V* increases (section S6), finding that for classical percolation

$$p_{\text{th}}≃1-\left(\text{ln}\frac{U}{U-1}\right)V^{-1}+O\left(V^{-2}\right)$$ (5)

and, for concurrence percolation

$$c_{\text{th}}≃1-\left(\frac{1}{4}\text{ln}V\right)V^{-1}+O\left(V^{-1}\text{ln}\text{ln}V\right)$$ (6)

The origin of the *V*^−1^ term is due to the fact that as *V* → ∞, loops within the (*U*, *V*) flower extend to infinite lengths, and the network essentially becomes tree-like (*46*). This suggests that nodes *A* and *B* are connected by only a single path. As a result, both thresholds approach 1 when *V* → ∞. However, for classical percolation, the prefactor of *V*^−1^ depends solely on *U*, signaling the significant role the shortest paths play in classical percolation near the critical threshold. In contrast, concurrence percolation shows a unique behavior. The presence of a logarithmic prefactor (1/4)ln*V* highlights the importance of the nonshortest paths in concurrence percolation. Moreover, this term is fully decoupled from *U*, suggesting that the shorter length scale no longer plays a determining role in *c*_th_. For different *U* = 2,5,10, on the one hand, the simulation results show the same conclusion as above. On the other hand, the analysis produces different constant limits for the classical case and different speed of going to zero for quantum case, exhibiting the necessity of the existence of shortest path *U* (section S6).

Our finding can also be illustrated as follows. At the percolation thresholds, the sponge-crossing connectivity along the longer path is given by $p_{\text{th}}^{V}≃\left(U-1\right)/U$ and $c_{\text{th}}^{V}≃V^{-1/4}$, respectively, when *V* → ∞ (section S6). In other words, for concurrence percolation, longer path connectivity tends to drop to zero ($c_{\text{th}}^{V}\to 0$) at the edge of dismantlement (*c* = *c*_th_), indicating that longer path connectivity must be exhausted as the network disintegrates in terms of concurrence. This contrasts with classical percolation, where the longer path connectivity is a nonzero constant ($p_{\text{th}}^{V}↛0$) at the edge of dismantlement (*p* = *p*_th_), meaning that the network can dismantle before longer paths connectivity are exhausted. Hence, the nonshortest paths play no role in the critical behavior of classical percolation.

The asymptotic behaviors of the critical exponents are also different. We analytically determine ν and *d*_*f*_ directly from the solution of *P*_SC_ and *P*_∞_ (cf. *C*_SC_ and *C*_∞_), and β from the hyperscaling relation *d*_*f*_ = *d* − β/ν. The results are summarized in Table 2 in the limit *V* → ∞. We find that in classical percolation, all dominant terms governing ν, *d* − *d*_*f*_, and β are determined by *U* only, once again signaling the dominance of the shorter paths. In contrast, in concurrence percolation, we notice the emergence of a very slow correction depending on the nonshortest paths (∼lnln*V*) affecting the dominant terms in ν and *d*_*f*_ (section S6). The corrections counterbalance each other in the calculation of β, resulting in a constant β = 1 that is independent on both *U* and *V*. Unexpectedly, this value coincides with the mean-field value of β for classical percolation (*45*). Because β is intrinsically tied to the definition of an order parameter, we hypothesize that this unique, constant value of β reveals an entirely distinct symmetry associated with the order parameter in concurrence percolation on (*U*, *V*) flowers.

### Superposition of paths in real-world networks

To demonstrate the generality of the results obtained on (*U*, *V*) flowers, we investigate the role of shortest and nonshortest paths in classical and quantum percolation in real networks. Specifically, we use path decomposition to systematically assess the contributions of shortest and nonshortest paths to overall network connectivity. It is worth noting that an *n*th generation (*U*, *V*) flower can be fully decomposed into 2^*n*^ nonoverlapping but intersecting paths between two boundary nodes *A* and *B* (Fig. 2C). In the language of graph theory, these paths are edge-disjoint, but not vertex-disjoint. Specifically, each of these paths can have a length that is given by one of the options $\left\{U^{n},U^{n-1}V,\ldots ,V^{n}\right\}$. The number of paths corresponding to length $U^{n-k}V^{k}$ is determined by the binomial coefficient $C_{n}^{k}\equiv \frac{n!}{k!\left(n-k\right)!}$.

The exact decomposition goes as follows. Initially, in the first generation, there are only two nonoverlapping paths between *A* and *B*, characterized by lengths *U* and *V*. Progressing to the second generation, each link from the previous generation is systematically replaced by the basic motif, necessitating a decision for each link within a previous path: to replace it by either a *U*-length path or a *V*-length path. Consequently, there are two nonoverlapping options: replacing all links entirely by *U*-length paths, or entirely by *V*-length paths. This bifurcation results in each earlier generation’s path splitting into two distinct paths. Thus, as a shorthand, each path can be encoded as a string of *n* characters (e.g., “UVVUUV...”), each designated as “U” or “V.” This string reflects the sequence of choices made at each generation, with U for choosing *U*-length paths and V for *V*-length paths. Each nonoverlapping path can be uniquely identified by such a string. The corresponding path length is *U*^*n*−*k*^*V*^*k*^, with *k* = 0, 1, …, *n*, representing the count of V’s in the string. For each possible length, there are $C_{n}^{k}$ such choices, which scale as $C_{n}^{k}≃{\left(2\mathrm{\pi k}\right)}^{-1/2}e^{k}k^{-k}n^{k}$ for *k* ≪ *n*, *k* → ∞, suggesting that nonshortest paths are exponentially more abundant.

**Fig. 4.**
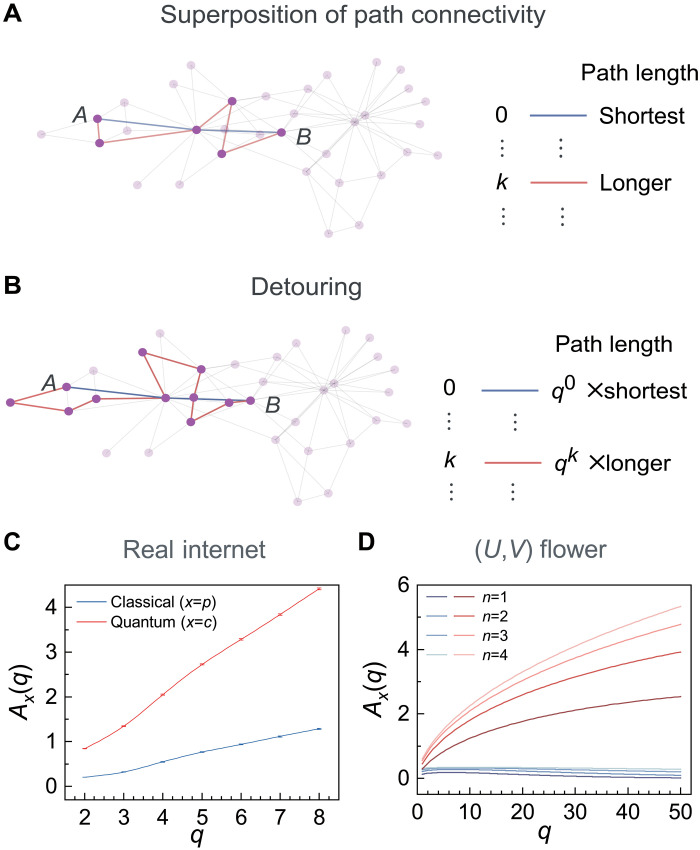
General path connectivity and detouring. (**A**) Superposition of paths in general networks. Nonoverlapping but potentially intersecting paths are selected between two hub nodes *A* and *B* and grouped by their lengths. (**B**) Detouring of longer paths in (A). Each path in group *k* is rerouted, becoming *q*^*k*^ times longer. The shortest path (blue line) is constant, while the red line shows an increased longer path when *q* = 2. (**C**) The anomalous resilience factors *A*_*p*_(*q*) and *A*_*c*_(*q*) show different scaling behaviors with respect to the detouring parameter *q* in the internet network topology (*19*). Higher *A*_*x*_(*q*) shows higher resilience to detouring. (**D**) Corresponding patterns are also observed in (*U*, *V*) flowers. As *q* increases, the classical $A_{p}\left(q\right)∼q^{0}$ (blue) approaches a constant value. In contrast, the quantum $A_{c}\left(q\right)∼\sqrt{q\text{ln}q/8}$ (red) increases with *q*.

The path decomposition is applicable to real networks with more general topologies. Specifically, given a real-world network, we select two nodes *A* and *B* and the nonoverlapping (but potentially intersecting) paths between them. These paths are ranked by their lengths and sequentially placed into different groups, labeled by *k* = 0,1,2, …, such that each successive group encompasses an exponentially larger number of longer paths (Fig. 4A).

We now consider the impact of detouring in the real-world network due to damages or congestion. Given that longer paths are more likely to be rerouted, we assume that the paths in group *k* increase their lengths by a factor of *q*^*k*^, where *q* ≥ 1 is a parameter that controls the extent of detouring. Meanwhile, the number of paths remains constant (Fig. 4B). To investigate the network’s resilience to detouring given the parameter *q*, we rewrite both *p*_th_ and *c*_th_ in terms of $\theta_{\text{th}}^{\left(p\right)}$ and $\theta_{\text{th}}^{\left(c\right)}$, expressed as

$$\theta_{\text{th}}^{\left(x\right)}\left(q\right)=\theta_{\text{th}}^{\left(x\right)}\left(\infty \right)-A_{x}\left(q\right)q^{-1}$$ (7)

where *q*^−1^ is a common factor, as we expect $A_{x}\left(q\right)q^{-1}\to 0$ when *q* → ∞ for both classical (*x* = *p*) and concurrence (*x* = *c*) percolation. We thus define $A_{x}\left(q\right)\equiv q\left[\theta_{\text{th}}^{\left(x\right)}\left(\infty \right)-\theta_{\text{th}}^{\left(x\right)}\left(q\right)\right]$ as the anomalous resilience factor, measuring the resilience of $\theta_{\text{th}}^{\left(x\right)}\left(q\right)$ against approaching the trivial threshold, $\theta_{\text{th}}^{\left(x\right)}\left(\infty \right)$. Note that, since $q^{0}\ll q^{k}$ for *k* > 0 in the *q* → ∞ limit, $\theta_{\text{th}}^{\left(x\right)}\left(\infty \right)$ will rely solely on the shortest path (*k* = 0).

While large-scale QNs are urgently needed, they are still in early stages, hindered by technological challenges in creating quantum memories (*47*, *48*). We here focus on existing internet networks, assuming QNs can follow the same topology. Recent experiments transmitting entanglement alongside classical signals via internet fibers confirm this potential (*49*, *50*). Here, we analyze a segment of the real-world internet at the autonomous-system level (*19*), representing data forwarding relationships among administrative entities or domains (section S7). Given two hub nodes and their nonoverlapping paths, detouring requires replacing each path in group *k* with a rerouted path from the same internet that is *q*^*k*^ longer. We determined $A_{x}\left(q\right)$ for *x* = *p*, *c* for various *q* (Fig. 4C).

For comparison, we also considered the (*U*, *V*) flower (Fig. 4D), where each path is lengthened by a factor of *q*^*k*^, yielding new path lengths of $\left\{U^{n},{\mathrm{qU}}^{n-1}V,\ldots ,q^{n}V^{n}\right\}=\left\{U^{n},\left(\mathrm{qV}\right)U^{n-1},\ldots ,{\left(\mathrm{qV}\right)}^{n}\right\}$, suggesting that *V* simply adjusts to *qV*. This suggests that the effect of lengthening the paths in the (*U*, *V*) flower can be achieved by simply adjusting the *V* parameter to *qV*. Therefore, rearranging Eqs. 5 and 6 leads to the theoretical predictions of the anomalous resilience factors for the (*U*, *V*) flower, $A_{p}\left(q\right)≃\text{ln}\left[U/\left(U-1\right)\right]/2=O\left(q^{0}\right)$, versus $A_{c}\left(q\right)≃V^{1/2}\sqrt{q\text{ln}q/8}$. The similarity of the dependencies of $A_{x}\left(q\right)$ on *q* between the internet and the (*U*, *V*) flower highlights a shared quantum characteristic: the faster increase of *A*_*x*_(*q*) for *x* = *c*, which indicates a higher resilience of concurrence connectivity. Our observation indicates the presence of such resilience in general network topologies, characterized by nonoverlapping but intersecting paths.

The distinct universality classes of classical and quantum percolation on (*U*, *V*) flowers, driven by their differing dependencies on nonshortest paths, suggest that these findings are broadly applicable to other network types, including real-world networks with complex connectivity patterns.

Note that given the very slow corrections ∼lnln*V* in the quantum critical exponents (Table 2), this extreme effect becomes observable only for a very large length scale, exceeding the internet diameter. However, if we consider “fundamental” networks, such as spin networks in loop quantum gravity (*51*), where the length unit is extremely small (the Planck scale 10^−35^ m), the corrections could potentially become detectable.

## DISCUSSION

In conclusion, our findings show the crucial role of nonshortest quantum entanglement paths in enhancing connectivity within QNs. This advancement builds on the theoretical breakthroughs achieved in previous studies of hierarchical scale-free network models (*23*, *24*). Unlike traditional lattice models, these innovative models allow for precise analytical analysis of the complex critical phenomena underlying QNs, marking a substantial step forward in the field.

From the theoretical, statistical physics perspective, our findings, which place concurrence percolation in a distinct universality class to classical percolation, rule out the possibility that differences between classical and concurrence percolation theories are confined merely to short-range (i.e., ultraviolet) details. Instead, variations stem from long-range (i.e., infrared) behaviors. This distinction emphasizes the fact that concurrence percolation represents a fundamentally different statistical framework compared to its classical counterpart. The question of whether concurrence percolation can be described by a field theory akin to the ϕ^3^ model (*52*) for classical percolation remains open. Such a “concurrence” field theory could simultaneously manifest two (or more) length scales, a common phenomenon in quantum critical systems (*53*). To this end, a crucial step involves defining a cluster-based order parameter (field) specific to concurrence percolation. Our non–cluster-based definition of the percolating strength *C*_∞_ on (*U*, *V*) flowers could potentially serve as a foundation for constructing a cluster-based order parameter.

From the perspective of quantum computation and communication, our research indicates that it is possible to achieve entanglement transmission via longer, exponentially weaker paths composed of partially entangled links, provided there is a compensatory increase in the number of such paths. This principle finds a parallel in branching processes, where infinite connectivity is achievable if the growing number of trees with a given size exactly compensates for the exponential decline in the probability of such a tree existing at the critical threshold (*40*). Further exploration of this concept through other models, such as the (*U*, *V*, *W*) flowers (with three different length scales), could provide deeper insights into the entanglement distribution efficiency in QNs. The principle of leveraging multiple nonshortest paths is particularly relevant to the rapid advancement of multiplexing techniques in quantum communication (*54*). However, the unavoidable mixing of entangled pure states with classical noise complexifies the scenario, underscoring the need for a hybrid model that incorporates elements of both classical and concurrence percolation. We expect that a stronger connectivity would still exist in mixed-state QNs, which, if feasible for use, must go beyond any completely classical treatments.

## Supplementary Materials

### This PDF file includes:

Sections S1 to S7 Figs. S1 to S9

## References

[1] S. Wehner, D. Elkouss, R. Hanson, Quantum internet: A vision for the road ahead. Science 362, eaam9288 (2018). 30337383 10.1126/science.aam9288

[2] S. Brito, A. Canabarro, R. Chaves, D. Cavalcanti, Statistical properties of the quantum internet. Phys. Rev. Lett. 124, 210501 (2020). 32530693 10.1103/PhysRevLett.124.210501

[3] J. Nokkala, J. Piilo, G. Bianconi, Complex quantum networks: A topical review. J. Phys. A Math. Theor. 57, 233001 (2024).

[4] E. Chitambar, M.-H. Hsieh, Relating the resource theories of entanglement and quantum coherence. Phys. Rev. Lett. 117, 020402 (2016). 27447493 10.1103/PhysRevLett.117.020402

[5] M. Caleffi, M. Amoretti, D. Ferrari, J. Illiano, A. Manzalini, A. S. Cacciapuoti, Distributed quantum computing: A survey. Comput. Netw. 254, 110672 (2024).

[6] C. H. Bennett, G. Brassard, C. Crepeau, R. Jozsa, A. Peres, W. K. Wootters, Teleporting an unknown quantum state via dual classical and Einstein-Podolsky-Rosen channels. Phys. Rev. Lett. 70, 1895–1899 (1993). 10053414 10.1103/PhysRevLett.70.1895

[7] D. Gottesman, Theory of quantum secret sharing. Phys. Rev. A 61, 042311 (2000).

[8] J. L. Park, The concept of transition in quantum mechanics. Found. Phys. 1, 23–33 (1970).

[9] S. Pirandola, R. Laurenza, C. Ottaviani, L. Banchi, Fundamental limits of repeaterless quantum communications. Nat. Commun. 8, 15043 (2017). 28443624 10.1038/ncomms15043 PMC5414096

[10] A. Acín, J. I. Cirac, M. Lewenstein, Entanglement percolation in quantum networks. Nat. Phys. 3, 256–259 (2007).

[11] X. Meng, J. Gao, S. Havlin, Concurrence percolation in quantum networks. Phys. Rev. Lett. 126, 170501 (2021). 33988406 10.1103/PhysRevLett.126.170501

[12] S. Galam, A. Mauger, Universal formulas for percolation thresholds. Phys. Rev. E 53, 2177–2181 (1996). 10.1103/physreve.53.2177 9964496

[13] R. Cohen, D. ben-Avraham, S. Havlin, Percolation critical exponents in scale-free networks. Phys. Rev. E 66, 036113 (2002). 10.1103/PhysRevE.66.036113 12366190

[14] H. Kesten, The critical probability of bond percolation on the square lattice equals 1/2. Commun. Math. Phys. 74, 41–59 (1980).

[15] X. Meng, X. Hu, Y. Tian, G. Dong, R. Lambiotte, J. Gao, S. Havlin, Percolation theories for quantum networks. Entropy 25, 1564 (2023). 37998256 10.3390/e25111564 PMC10670322

[16] O. Malik, X. Meng, S. Havlin, G. Korniss, B. K. Szymanski, J. Gao, Concurrence percolation threshold of large-scale quantum networks. Commun. Phys. 5, 193 (2022).

[17] C. Song, S. Havlin, H. A. Makse, Origins of fractality in the growth of complex networks. Nat. Phys. 2, 275–281 (2006).

[18] L. K. Gallos, C. Song, S. Havlin, H. A. Makse, Scaling theory of transport in complex biological networks. Proc. Natl. Acad. Sci. U.S.A. 104, 7746–7751 (2007). 17470793 10.1073/pnas.0700250104 PMC1876518

[19] G. Tilch, T. Ermakova, B. Fabian, A multilayer graph model of the internet topology. IJNVO 22, 219–245 (2020).

[20] Z. Wang, D. Luo, O. Cats, T. Verma, Unraveling the hierarchy of public transport networks, in *2020 IEEE 23rd International Conference on Intelligent Transportation Systems (ITSC)* (IEEE, 2020), pp. 1–6.

[21] P. Moretti, M. A. Muñoz, Griffiths phases and the stretching of criticality in brain networks. Nat. Commun. 4, 2521 (2013). 24088740 10.1038/ncomms3521

[22] O. Sporns, G. Tononi, R. Kötter, The human connectome: A structural description of the human brain. PLOS Comput. Biol. 1, e42 (2005). 16201007 10.1371/journal.pcbi.0010042 PMC1239902

[23] H. D. Rozenfeld, S. Havlin, D. ben-Avraham, Fractal and transfractal recursive scale-free nets. New J. Phys. 9, 175 (2007).

[24] H. D. Rozenfeld, D. ben-Avraham, Percolation in hierarchical scale-free nets. Phys. Rev. E Stat. Nonlin. Soft Matter Phys. 75, 061102 (2007). 17677215 10.1103/PhysRevE.75.061102

[25] G. Dong, F. Wang, L. M. Shekhtman, M. M. Danziger, J. Fan, R. Du, J. Liu, L. Tian, H. E. Stanley, S. Havlin, Optimal resilience of modular interacting networks. Proc. Natl. Acad. Sci. U.S.A. 118, e1922831118 (2021). 34035163 10.1073/pnas.1922831118 PMC8179239

[26] J. Gao, B. Barzel, A.-L. Barabási, Universal resilience patterns in complex networks. Nature 530, 307–312 (2016). 26887493 10.1038/nature16948

[27] X. Liu, D. Li, M. Ma, B. K. Szymanski, H. E. Stanley, J. Gao, Network resilience. Phys. Rep. 971, 1–108 (2022).

[28] S. Pirandola, End-to-end capacities of a quantum communication network. Commun. Phys. 2, 51 (2019).

[29] H. Kobayashi, F. Le Gall, H. Nishimura, M. Rötteler, Perfect quantum network communication protocol based on classical network coding, in *2010 IEEE International Symposium on Information Theory* (IEEE, 2010), pp. 2686–2690.

[30] S. Boettcher, V. Singh, R. M. Ziff, Ordinary percolation with discontinuous transitions. Nat. Commun. 3, 787 (2012). 22510692 10.1038/ncomms1774

[31] G. Bianconi, R. M. Ziff, Topological percolation on hyperbolic simplicial complexes. Phys. Rev. E 98, 052308 (2018).

[32] G. Bianconi, I. Kryven, R. M. Ziff, Percolation on branching simplicial and cell complexes and its relation to interdependent percolation. Phys. Rev. E 100, 062311 (2019). 31962446 10.1103/PhysRevE.100.062311

[33] I. Kryven, R. M. Ziff, G. Bianconi, Renormalization group for link percolation on planar hyperbolic manifolds. Phys. Rev. E 100, 022306 (2019). 31574679 10.1103/PhysRevE.100.022306

[34] H. Sun, R. M. Ziff, G. Bianconi, Renormalization group theory of percolation on pseudofractal simplicial and cell complexes. Phys. Rev. E 102, 012308 (2020). 32795074 10.1103/PhysRevE.102.012308

[35] G. Bianconi, *Higher-Order Networks* (Cambridge Univ. Press, 2021).

[36] M. Gromov, Hyperbolic groups, in *Essays in Group Theory* (Springer, 1987), pp. 75–263.

[37] R. J. Duffin, Topology of series-parallel networks. J. Math. Anal. Appl. 10, 303–318 (1965).

[38] J. C. Wierman, Bond percolation on honeycomb and triangular lattices. Adv. Appl. Probab. 13, 298–313 (1981).

[39] J. Cardy, Conformal invariance and percolation. arXiv:0103018 (2001).

[40] K. Christensen, N. R. Moloney, *Complexity and Criticality* (World Scientific Publishing Company, 2005), vol. 1.

[41] L. Versfeld, Remarks on star-mesh transformation of electrical networks. Electron. Lett. 6, 597–599 (1970).

[42] W. Chen, J. Nagler, X. Cheng, X. Jin, H. Shen, Z. Zheng, R. M. D’Souza, Phase transitions in supercritical explosive percolation. Phys. Rev. E 87, 052130 (2013). 10.1103/PhysRevE.87.052130 23767510

[43] R. M. D’Souza, J. Gómez-Gardeñes, J. Nagler, A. Arenas, Explosive phenomena in complex networks. Adv. Phys. 68, 123–223 (2019).

[44] D. Stauffer, A. Aharony, *Introduction to Percolation Theory: Second Edition* (CRC Press, 2018).

[45] D. ben-Avraham, S. Havlin, *Diffusion and Reactions in Fractals and Disordered Systems* (Cambridge Univ. Press, ed. 3, 2010).

[46] A. W. Sandvik, Ground states of a frustrated quantum spin chain with long-range interactions. Phys. Rev. Lett. 104, 137204 (2010). 20481910 10.1103/PhysRevLett.104.137204

[47] C. Simon, M. Afzelius, J. Appel, A. B. de la Giroday, S. J. Dewhurst, N. Gisin, C. Y. Hu, F. Jelezko, S. Kröll, J. H. Müller, J. Nunn, E. S. Polzik, J. G. Rarity, H. De Riedmatten, W. Rosenfeld, A. J. Shields, N. Sköld, R. M. Stevenson, R. Thew, I. A. Walmsley, M. C. Weber, H. Weinfurter, J. Wrachtrup, R. J. Young, Quantum memories. Eur. Phys. J. 58, 1–22 (2010).

[48] X. Meng, N. Lo Piparo, K. Nemoto, I. A. Kovács, Quantum networks enhanced by distributed quantum memories. arXiv:2403.16367 [quant-ph] (2024).

[49] J. Chung, E. M. Eastman, G. S. Kanter, K. Kapoor, N. Lauk, C. H. Pena, R. K. Plunkett, N. Sinclair, J. M. Thomas, R. Valivarthi, S. Xie, R. Kettimuthu, P. Kumar, P. Spentzouris, M. Spiropulu, Design and implementation of the Illinois Express Quantum Metropolitan Area Network. IEEE Trans. Quantum Eng. 3, 1–20 (2022).

[50] J. M. Thomas, G. S. Kanter, P. Kumar, Designing noise-robust quantum networks coexisting in the classical fiber infrastructure. Opt. Express 31, 43035–43047 (2023). 38178406 10.1364/OE.504625

[51] A. Ashtekar, E. Bianchi, A short review of loop quantum gravity. Rep. Prog. Phys. 84, 042001 (2021). 10.1088/1361-6633/abed91 33691292

[52] D. J. Amit, Renormalization of the Potts model. J. Phys. A: Math. Gen. 9, 1441–1459 (1976).

[53] H. Shao, W. Guo, A. W. Sandvik, Quantum criticality with two length scales. Science 352, 213–216 (2016). 26989196 10.1126/science.aad5007

[54] N. Lo Piparo, M. Hanks, K. Nemoto, W. J. Munro, Aggregating quantum networks. Phys. Rev. A 102, 052613 (2020).

[55] G. Bianconi, Statistical mechanics of multiplex networks: Entropy and overlap. Phys. Rev. E 87, 062806 (2013). 10.1103/PhysRevE.87.062806 23848728

