# Supplementary material for: Unveiling the importance of nonshortest paths in quantum networks
Source: Sci Adv. 2025 Feb 26;11(9):eadt2404. doi: 10.1126/sciadv.adt2404 (PMC11864168; doi:10.1126/sciadv.adt2404)
Supplement: Supplementary file 1 — Sections S1 to S7 Figs. S1 to S9 [file sciadv.adt2404_sm.pdf]

Science Advances
AAAS

Supplementary Materials for

Unveiling the importance of nonshortest paths in quantum networks

Xinqi Hu *et al.*

Corresponding author: Gaogao Dong, dfocus.gao@gmail.com;
Renaud Lambiotte, renaud.lambiotte@maths.ox.ac.uk; Xiangyi Meng, xmenggroup@gmail.com;
Kim Christensen, k.christensen@imperial.ac.uk

Sci. Adv. **11**, eadt2404 (2025)
DOI: 10.1126/sciadv.adt2404

This PDF file includes:

Sections S1 to S7
Figs. S1 to S9

S1 Non-hyperbolicity of (U, V) flowers

Gromov's δ-hyperbolicity (*35, 36*) provides a convenient quantification of the overall hyperbolicity of a network. It is defined as follows (*35*): A network is said to be δ-hyperbolic if there exists a finite δ such that for any triplet of nodes r, s, and q connected by the shortest paths $\mathcal{P}_{rs}$, $\mathcal{P}_{sq}$, and $\mathcal{P}_{rq}$ respectively, the union of the δ-neighborhood of any pair of shortest paths includes all nodes that belong to the third shortest path.

Based on this definition, we will show that when $U > 1$, the (U, V) flowers are not δ-hyperbolic for any finite δ.

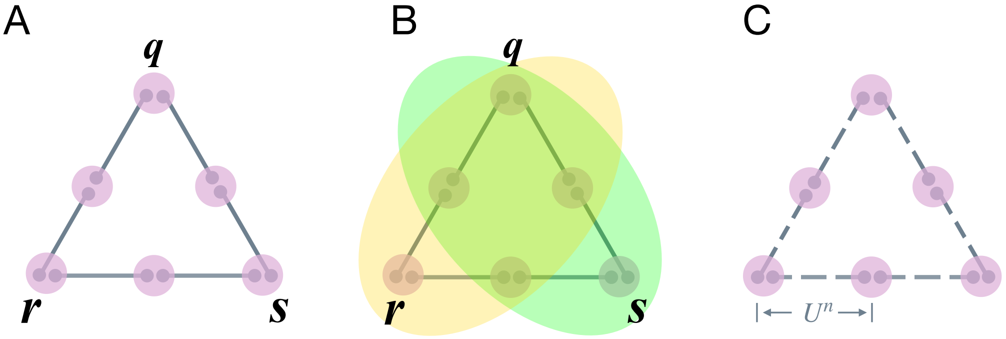

Figure S1: **Proof of non-hyperbolicity of (U, V) flower for $U > 1$.** (**A**) (U, V) flower with $U = 2$, $V = 4$ of generation $n = 1$. Three chosen nodes q, r, s are positioned at a distance of 2. (**B**) By choosing $\delta = 1$, the δ-neighborhood of the paths $\mathcal{P}_{rq}$ and $\mathcal{P}_{sq}$ (yellow and green shades) includes all the nodes in the third path, $\mathcal{P}_{rs}$. (**C**) In the $(n + 1)$-th generation, each link (solid line) in (**A**) is replaced by an n-th generation of the (U, V) flower (dashed line) by iteration. This means that the shortest distance between r and s in (**A**) changes from 2 to $2U^n$, which is much greater than 2 for large n and $U > 1$.

Let us consider three nodes r, s, q in the (U, V) flower at generation $n = 1$, such that the three nodes are equidistant, each separated by a distance of $(U + V)/3$. As a result, the corresponding three shortest paths $\mathcal{P}_{rs}$, $\mathcal{P}_{rq}$, and $\mathcal{P}_{sq}$ do not overlap with each other (Fig. S1A). In order to include nodes belonging to the third path, say $\mathcal{P}_{rs}$, within the δ-neighborhood of the union of the other two paths $\mathcal{P}_{rq}$ and $\mathcal{P}_{sq}$, δ must satisfy

$$\delta \geq \max_{i \in \mathcal{P}_{rs}} \left\{ \min \left\{ \min_{j \in \mathcal{P}_{rq}} d(i, j), \min_{k \in \mathcal{P}_{sq}} d(i, k) \right\} \right\}, \tag{S1}$$

where $d(i, j)$ indicates the distance between node i and j. Since the shortest path between node pairs in $\mathcal{P}_{rs}$ and $\mathcal{P}_{qr}$ (or $\mathcal{P}_{qs}$) must pass through node q (or s), therefore,

$$\min_{j \in \mathcal{P}_{rq}} d(i, j) = d(i, r), \quad \min_{k \in \mathcal{P}_{sq}} d(i, k) = d(i, s). \tag{S2}$$

When i is the midpoint of r and s, the quantity on the right-hand side of Eq. (S1) is maximized, which is equal to (Fig. S1B)

$$\delta \geq \max_{i \in \mathcal{P}_{rs}} \{\min \{d(i, r), d(i, s)\}\} = \frac{1}{2}\frac{U + V}{3}. \tag{S3}$$

Moving to the $(n + 1)$-th generation, since every two neighboring nodes in the first-generation (U, V) flower will now be separated by a shortest distance U^n (Fig. S1C), δ at the $(n + 1)$-th generation must satisfy

$$\delta \geq \frac{1}{2}\frac{U + V}{3}U^n, \tag{S4}$$

which is unbounded at $n \longrightarrow \infty$. Thus, general (U, V) flowers with $U > 1$ are not hyperbolic. For the special case of $U = 1$, the (U, V) flowers reduce to the pseudofractal simplicial and cell complexes, which have been shown to be hyperbolic (*35*).

S2 Critical conditions of classical and concurrence percolation transitions

The critical threshold p_{th} (c_{th}) can be obtained by analyzing the stability of the fixed points $x = x^*$ that satisfy the exact renormalization-group (RG) equation:

$$\mathcal{R}(x) = x \tag{S5}$$

where

$$\mathcal{R}(x) = \text{para}(\text{seri}(\overbrace{x, x, \ldots, x}^{U}), \text{seri}(\overbrace{x, x, \ldots, x}^{V})). \tag{S6}$$

Here, for simplicity, we denote both the probability p and the concurrence c by x. The stability can be determined by the derivative of $\mathcal{R}(x)$ at $x = x^*$. If $|\mathcal{R}'(x^*)| < 1$, the fixed point x^* is stable; if $|\mathcal{R}'(x^*)| > 1$, the fixed point x^* is unstable. We will show that both classical and concurrence "quantum" percolation on (U, V) flowers have only one critical threshold p_{th} (or c_{th}).

We have, for classical percolation,

$$\mathcal{R}(p) = 1 - \left(1 - p^U\right)\left(1 - p^V\right); \tag{S7}$$

and for concurrence percolation,

$$\mathcal{R}(c) = \sqrt{1 - (2K(c) - 1)^2}, \tag{S8}$$

where

$$K(c) = \max\left(\frac{1 + \sqrt{1 - c^{2U}}}{2}\frac{1 + \sqrt{1 - c^{2V}}}{2}, \frac{1}{2}\right). \tag{S9}$$

When $U = 1$, for both classical and concurrence percolation, $\mathcal{R}(x) = x$ only has two trivial fixed points $x^* = 0$ and $x^* = 1$, and only $x^* = 1$ is stable. This indicates a critical threshold $x_{\text{th}} = 0$. When $U > 1$, for both classical and concurrence percolation, $\mathcal{R}(x) = x$ has three fixed points: $x^* = 0$, $x^* = 1$, and a nontrivial fixed point $0 < x^* < 1$ (Figs. S2A and S2B).

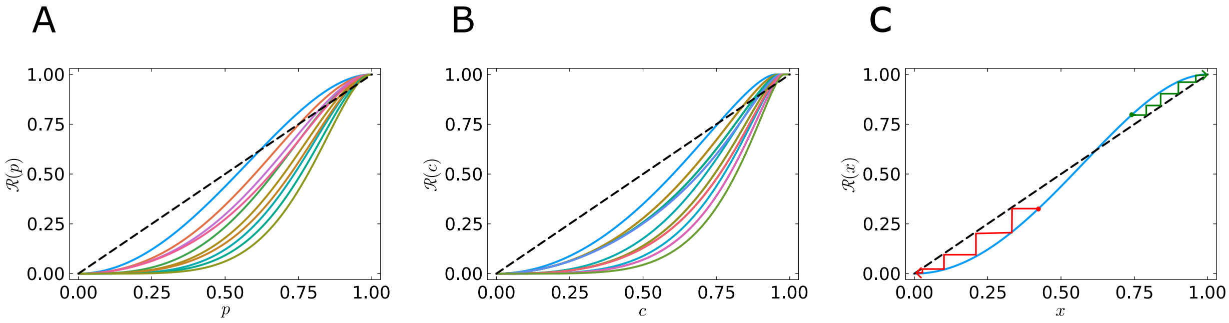

Figure S2: **Illustration of fixed points of $\mathcal{R}(x) = x$ and their stability.** (**A**) The map $\mathcal{R}(p)$ defined in Eq. (S7) as a function of p. (**B**) $\mathcal{R}(c)$ defined in Eq. (S8) as a function of c. Both maps are shown for $U, V = 2, 3, 4, 5$. (**C**) Cobweb plot illustrating the stability of the fixed points. For any $x < x_{\text{th}}$ (the red point), $x \in \{c, p\}$, the iterative map will converge to zero, while for any $x > x_{\text{th}}$, the iterative map will converge to one. The iterative calculations are graphically shown as the red and green lines.

We observe that $x^* = 0$ and $x^* = 1$ are two stable fixed points, since $\mathcal{R}'(0) = \mathcal{R}'(1) = 0$, and the non-trivial fixed point is always unstable (Fig. S2C). Thus, the nontrivial fixed point gives the critical threshold x_{th}: iterating Eq. (S6) infinite times yields either 0 for $x < x_{\text{th}}$ or 1 for $x > x_{\text{th}}$. Therefore, the percolation threshold for both classical and concurrence percolation is unique.

S3 A non-cluster definition of percolating strength

First, we categorize the nodes in the (U, V) flower into different layers based on the generation in which they were introduced into the network (Figs. S3A and S3B). The deepest layer consists of all nodes with the lowest degree of 2, representing the nodes introduced in the last generation. Note that the number of nodes introduced in the (U, V) flower increases exponentially with each generation. Consequently, the probability that a node in the deepest layer connects to A or B governs *the probability of a randomly chosen node in the bulk to reach the boundaries (A or B) of the network*, which denotes the strength of the percolating cluster, P_∞ (*23*).

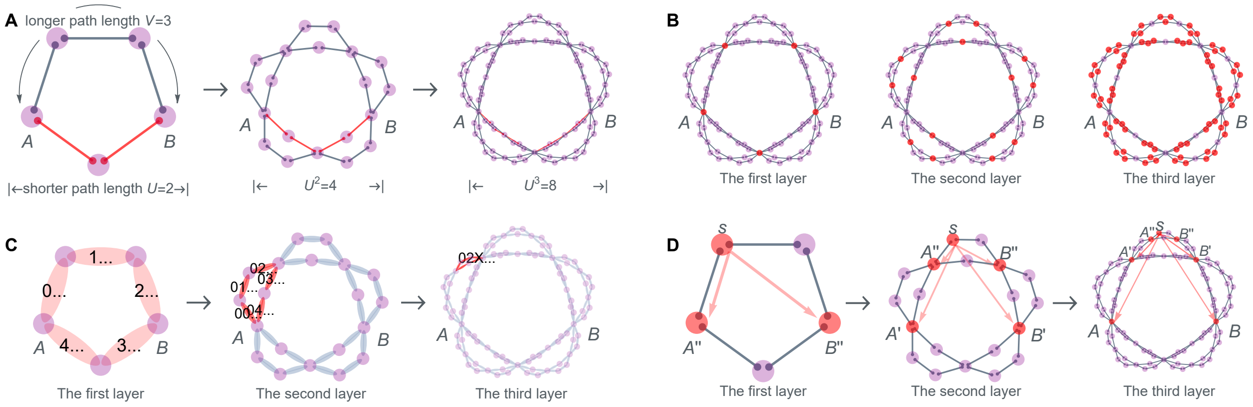

Figure S3: **Hierarchical scale-free networks and non-cluster definition of P_∞.** (**A**) The construction of hierarchical scale-free quantum networks (U, V) flowers where $U > 1$ is the shorter length scale while $V \geq U$ is the longer length scale. (**B**) Nodes on different layers. The deepest (third) layer has the most number of nodes. (**C**) A node has the same probability of belonging to one of these $(U + V)$ regimes, for each layer. (**D**) We can find a transfer matrix demonstrating the probability that a node connecting to A' (or B') at layer 2 in terms of the probability that this node connecting to A'' (or B'') at layer 1, or the probability that it connecting to A (or B) at layer 3 in terms of the probability that this node connecting to A' (or B') at layer 2.

However, not all nodes at the deepest layer are equivalent: some are closer to A (or B) and some are farther. When $n \to \infty$, each node at the deepest layer can be characterized by an infinite series of $(U + V)$ digits: For the $(2, 3)$ flower, for example, each node is characterized by, e.g., $434032\ldots$, where the n-th digit represents which link $(0, 1, 2, 3, 4)$ at the n-th generation the node traces back to and originates from (Fig. S3C). The probability that the node at position, e.g., $434032\ldots$,

connects to A (or B) is denoted by $P_{434032...}$, which is a 2×1 vector: (prob. to A, prob. to $B)^T$. The probability of randomly choosing a node at the deepest layer that connects to A (or B) is, therefore, $P_\infty = P_{0000...} + P_{1000...} + P_{2000...} + \cdots + P_{4340...} + \ldots$ which is also represented by a 2×1 vector.

Now, what is special about classical percolation in the (U, V) flower is that $P_{434032...}$ can indeed be written as the multiplication of a series of 2×2 matrices and a trivial vector $(1, 0)^T$ (the exact form of the trivial vector should not matter): $P_{434032...} = (P_4 P_3 P_4 P_0 P_3 P_2 \ldots) (1, 0)^T$. Each matrix (e.g., P_0) is a function of p. The four entries of P_0 correspond to the conditional probabilities of a node connecting to A (B) at the n-th layer given the probability of the same node connecting to A' (B') at the $(n+1)$-th layer (Fig. S3D). In other words, P_0 plays the role of the transfer matrix in statistical physics, converting from the $(n+1)$-th to the n-th layer, provided that the node traces back to link 0 at the $(n+1)$-th generation. By averaging over the five possible digits $(0, 1, 2, 3, 4)$, we can rewrite P_∞ as:

$$\begin{aligned} P_\infty &= [\frac{P_0 + P_1 + P_2 + P_3 + P_4}{5}][\frac{P_0 + P_1 + P_2 + P_3 + P_4}{5}] \ldots \\ &= \bar{P}\bar{P} \ldots \end{aligned} \tag{S10}$$

This allows us to solve β accurately, which is derived from the largest eigenvalue of $\bar{P} = (P_0 + P_1 + P_2 + P_3 + P_4)/5$. The result is identical to Rozenfeld and ben-Avraham's calculation (*24*).

What complicates the calculation for concurrence percolation is that we cannot write down $C_{434032...}$ in terms of a series of transfer matrices. Instead, we write down $C_{434032...}$ in terms of a series of transfer functions, given by $C_{434032...} = C_4(C_3(C_4(C_0(C_3(C_2))))) \ldots$ where each function (e.g., C_0) has 2×1 input and 2×1 output. The exact form of the function depends on not only the series and parallel rules but also higher-order rules.

Now, we may nominally define the percolating strength as

$$C_\infty = \frac{C_0 + C_1 + C_2 + C_3 + C_4}{5}(\frac{C_0 + C_1 + C_2 + C_3 + C_4}{5}(\ldots)). \tag{S11}$$

Here, $[(C_0 + C_1 + C_2 + C_3 + C_4)/5](\cdot)$ denotes the average of the output values of the five different functions, $C_0(\cdot), \ldots, C_4(\cdot)$. This allows us to simulate C_∞ for n layers, arriving at a numerical value of β by fitting to the power law.

S4 Star-mesh transform

The challenge of using Eq. (S10) or Eq. (S11) to calculate the non-cluster-defined percolation strength is that the matrices $P_0 \ldots$ (or the functions $C_0 \ldots$) depend not only on the series and parallel rules but also on higher-order connectivity rules, which cannot be decomposed into series and parallel rules (*11*). For classical percolation, the higher-order connectivity rules are known, but are highly complicated; for concurrence percolation, these rules are simply unknown. This calls for an approximating approach to treat the higher-order rules as approximate combinations of series and parallel rules only. The star-mesh transform serves for this purpose.

S4.1 Definition

A star-mesh transform (*41*) can establish a local equivalence of the connectivity between an $(s+1)$-node star graph and an s-node complete graph (Fig. S4A) based only on series and parallel rules (*11*). We denote the $(s+1)$-node star graph as $\mathcal{G}(s)$, with one root node and s leaf nodes where the weight of the i-th link is θ_i. Correspondingly, the star-mesh transform of $\mathcal{G}(s)$, i.e., the s-node complete graph, is denoted as $\mathcal{G}'(s)$, where the weights of the $s(s-1)/2$ links are assumed as $(\theta_{12}, \theta_{13}, \ldots, \theta_{1s}, \ldots, \theta_{s-1,s})$. The equivalence between $\mathcal{G}(s)$ and $\mathcal{G}'(s)$ are given by $s(s-1)/2$ independent equations:

$$
\begin{aligned}
\mathrm{seri}\left(\theta_1, \theta_2\right) &= \mathrm{cross}\left(1, 2; \mathcal{G}'\left(s\right)\right), \\
\mathrm{seri}\left(\theta_1, \theta_3\right) &= \mathrm{cross}\left(1, 3; \mathcal{G}'\left(s\right)\right), \\
&\ldots \\
\mathrm{seri}\left(\theta_1, \theta_s\right) &= \mathrm{cross}\left(1, s; \mathcal{G}'\left(s\right)\right), \\
&\ldots \\
\mathrm{seri}\left(\theta_{s-1}, \theta_s\right) &= \mathrm{cross}\left(s-1, s; \mathcal{G}'\left(s\right)\right).
\end{aligned}
\tag{S12}
$$

The expression $\mathrm{seri}(\theta_i, \theta_j)$ here is the series-sum (based on the series rule) of the i-th and j-th links in $\mathcal{G}(s)$, and $\mathrm{cross}(i, j; \mathcal{G}'(s))$ is the net weight across the two nodes i and j in $\mathcal{G}'(s)$ and can be calculated by recursively degrading $\mathcal{G}'(s)$ to a link between i and j.

Particularly, first we arbitrarily choose a node (except i or j) from $\mathcal{G}'(s)$ as the root of an extracted sub-star-graph $(\mathrm{sub}\mathcal{G}')(s-1)$ which has $s-1$ links connected to the root. Using the star-mesh

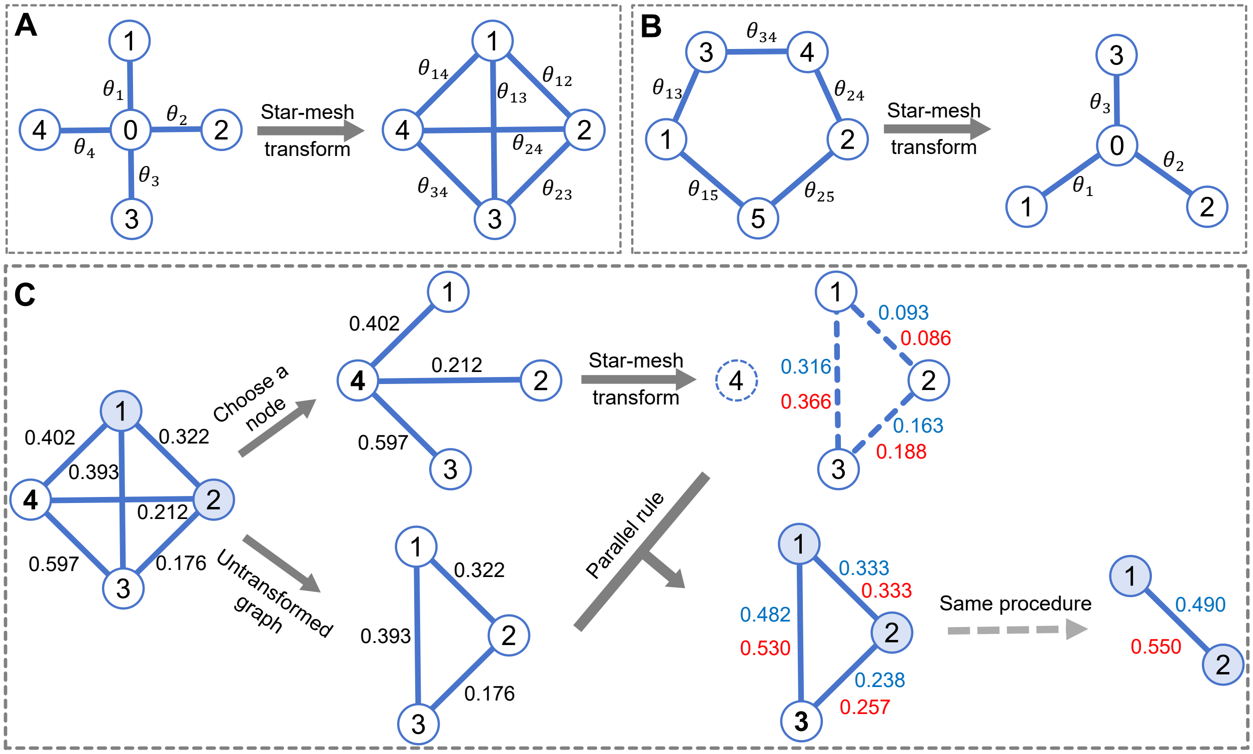

Figure S4: **Star-mesh transform.** (**A**) Star-mesh transform from an $(s+1)$-node star graph to an s-node complete graph. Applying series and parallel rules recursively, the link weights are determined by solving a double-recursive system of $s(s-1)/2$ equations. (**B**) Star-mesh transform from a graph with loop to a star graph. The final weight between same two nodes of the two graphs should be consistent. (**C**) Detailed procedure of solving the net weight across, e.g., nodes 1 and 2, by reducing the $(s-1)$-node complete graph (blue: classical percolation; red: concurrence percolation).

transform, this sub-star-graph are mapped into an $(s-1)$-complete graph denoted by $(\text{sub}\mathcal{G}')'(s-1)$. Then we combine it (based on the parallel rule) with the complement subgraph of $\mathcal{G}'(s)$, denoted by $\mathcal{G}'(s) \setminus (\text{sub}\mathcal{G}')(s-1)$, getting a new $(s-1)$-complete graph. We define the new graph as $\text{Comb}\left(\mathcal{G}_\alpha, \mathcal{G}_\beta\right)$, which has each of its link weight given by $\theta_{ij} = \text{para}(\alpha_{ij}, \beta_{ij})$ with $\alpha_{ij} \in \mathcal{G}_\alpha$ and $\beta_{ij} \in \mathcal{G}_\beta$. Thus, the connectivity between i and j in $\mathcal{G}'(s)$ can be calculated by

$$\text{cross}(i, j; \mathcal{G}'(s)) = \text{cross}(i, j; \text{Comb}\left((\text{sub}\mathcal{G}')'(s-1), \mathcal{G}'(s) \setminus (\text{sub}\mathcal{G}')(s-1)\right)). \tag{S13}$$

Next, we choose the nodes of $\mathcal{G}'(s)$ one after the other and operate the same process, until only nodes i and j and a link between them are left, finally producing a function of $\text{cross}(i, j; \mathcal{G}'(s))$ related to $(\theta_1, \ldots, \theta_s)$. The entire procedure involves a $(s-1)$-level star-mesh transform, thus

is a double recursion (Fig. S4C). Since for concurrence percolation, a closed-form solution of Eqs. (S12) have not been found, we used the Broyden's root-finding algorithm to numerically find the $s(s-1)/2$ weights θ_{ij} that satisfy Eqs. (S12).

The star-mesh transform is similar to the real-space renormalization group (RG) for percolation theory, but is more general and suits for any networks, not just lattices. By consecutively applying it on transforming a star graph to a complete graph where a node is reduced for each time, which allows the degradation of network structure, the connectivity between two nodes A and B can be well approximated by the final weight θ of the link between them.

Alternatively, it can also be used to convert a cycle to a star graph. Based on the connectivity between same two nodes i and j, the link weights of this transformed star graph $(\theta_1, \theta_2, \ldots)$ is solved by the equations with the weights of the original graph. For example, in Fig. S4B, the group of equations is:

$$\begin{aligned} \mathrm{seri}\,(\theta_1, \theta_2) &= \mathrm{para}\,(\mathrm{seri}\,(\theta_{15}, \theta_{25})\,, \mathrm{seri}\,(\theta_{13}, \theta_{34}, \theta_{24}))\,, && \text{(S14a)} \\ \mathrm{seri}\,(\theta_1, \theta_3) &= \mathrm{para}\,(\theta_{13}, \mathrm{seri}\,(\theta_{15}, \theta_{25}, \theta_{24}, \theta_{34}))\,, && \text{(S14b)} \\ \mathrm{seri}\,(\theta_2, \theta_3) &= \mathrm{para}\,(\mathrm{seri}\,(\theta_{24}, \theta_{34})\,, \mathrm{seri}\,(\theta_{13}, \theta_{15}, \theta_{25}))\,. && \text{(S14c)} \end{aligned}$$

We will use the star-mesh transform to calculate and compare the non-cluster-defined P_∞ and C_∞ in the following sections.

S4.2 Validity of the star-mesh transform

Since for classical percolation, the exact cluster-defined P_∞ is known, this allows us to calculate the non-cluster-defined, classical P_∞ using the star-mesh transform for different network topologies, testing the validity of the approach by comparing to exact results.

Firstly, we compare the exact result of classical percolation [$\beta = 0.165\ldots$ for $(2,2)$ flowers] with the star-mesh-transform result of classical percolation [$\beta \approx 0.168829(3)$, which is approximate]. Considering that the star-mesh transform is a type of RG, the result is very plausible for investigating ordinary second-order phase transitions.

Next, to further assess the accuracy and generality of the star-mesh transform, here we extend to hyperbolic networks, focusing on the $(1, V)$ flowers which, as noted in Section S1, are hyperbolic and differ from general (U, V) flowers with $U > 1$. It is worth noting that $(1, V)$ flowers can

be equivalently treated as pseudofractal simplicial and cell complexes formed by $(V+1)$-sided polygons (*34*). This enables a cluster-based, analytical calculation of the peculiar critical behavior of P_∞ near the trivial critical threshold, $p_{\text{th}} = 0$. It can be shown that the critical behavior follows a special scaling form (*34*):

$$P_\infty \propto (p - p_{\text{th}}) \exp\left[-\alpha/(p - p_{\text{th}})^{V-1}\right]. \tag{S15}$$

In Fig. S5, instead of using the cluster-based definition of P_∞, we calculate P_∞ for $(1, V)$ flowers of different V and system size N using the star-mesh transform (colored lines) and compare the numerical results with the exact scaling form, Eq. (S15) (dashed line). Notably, the star-mesh transform results asymptotically agree with the scaling form. In addition, the finite-size effect of the saturation of P_∞ for $p \to p_{\text{th}}$ (or $-\ln p \to +\infty$, as in Fig. S5) matches our theoretical understanding (*34*), suggesting high accuracy of our star-mesh transform method.

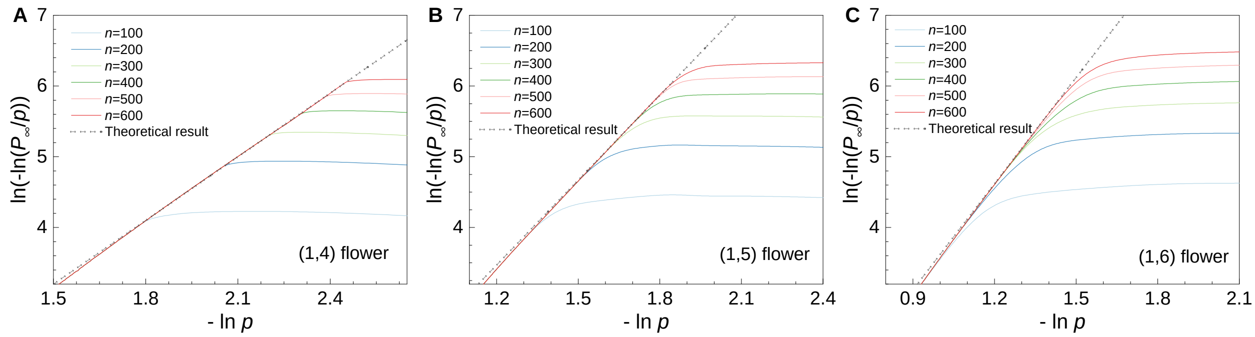

Figure S5: **Critical scaling of P_∞ as a function of p for $(1, V)$ flowers.** This figure shows the critical behavior following a special scaling of $P_\infty \propto p \exp(-\alpha/p^{V-1})$ yielding $\ln(-\ln(P_\infty/p)) = \ln\alpha - (V-1)\ln p$ (see Ref. (*34*)) for different (**A**) $V = 4$, (**B**) $V = 5$ and (**C**) $V = 6$. In infinite network limit (dashed line), the scaling is predicted by analyzing the critical behavior of system size $N \sim (V+1)^n$ with $n = 100, 200, \ldots, 600$.

We further apply the star-mesh transform to the Farey graphs, another type of hyperbolic networks (*30*). At the critical threshold, $p_{\text{th}} = 1/2$, the percolating strength has been shown to follow (*30*)

$$N_g \equiv NP_\infty = N^{d_f(p)/d}, \tag{S16}$$

where the fractal dimension of the percolating cluster now depends on p:

$$d_f(p)/d \simeq 1 - \frac{8(p - p_{\text{th}})^2}{} \tag{S17}$$

near $p \to p_{\text{th}}^{-}$.

In Fig. S6, we calculate P_∞ for different N, again using the non-cluster definition based on the star-mesh transform. Here, again, we observe not only the asymptotic agreement of the scaling form [Eq. (S17)] but also the correct finite-size effect.

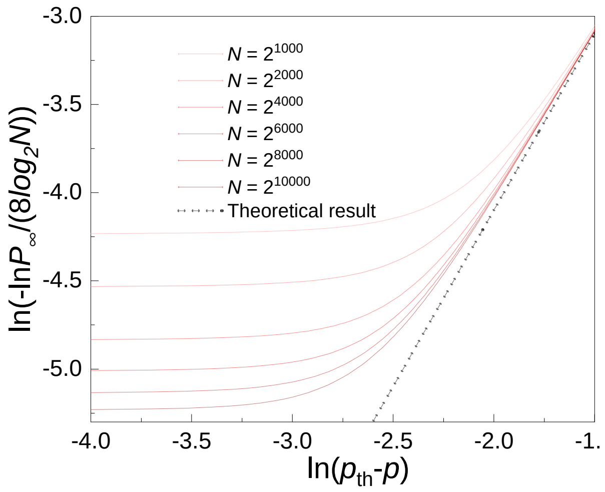

Figure S6: **Finite size scaling of P_∞ as a function of $p_{\text{th}} - p$.** Red lines from lightest to darkest represent network size from $N = 2^{1000}$ to $N = 2^{10000}$. Dashed line denotes the theoretical prediction $P_\infty = N^{d_f(p)/d-1}$, where $d_f(p)/d - 1 \simeq -8(p - p_{\text{th}})^2/\ln 2$.

Taken together, the star-mesh transform is not only useful for calculating the non-cluster definition of percolation strength for ordinary second-order phase transitions but also versatile enough for investigating more peculiar critical behaviors. This will be crucial for studying concurrence percolation (where clusters are not well defined) in hyperbolic networks in the future.

S5 Hyperscaling relation test

The alternative, non-cluster definition of P_∞ and C_∞ leads to a numerical method of calculating the critical exponents. Here we focus on $(2, 2)$ flowers. Let $L \to \infty$, it can be easily derived from the traditional relations. More intuitively, for classical percolation $1/\nu \simeq -\partial(|p_{\text{th}}(L) - p_{\text{th}}|)/\partial L$ yielding $1/\nu \approx 0.61152$ while for concurrence percolation $1/\nu \simeq -\partial(|c_{\text{th}}(L) - c_{\text{th}}|)/\partial L$ yielding $1/\nu \approx 0.73911$ (Figs. S7A–D), which are in very good agreement with the analytical result. And $\beta \simeq -\nu\partial \ln P_\infty/\partial \ln L$ or $\beta \simeq -\nu\partial \ln C_\infty/\partial \ln L$ (Figs. S7E–F), while $d_f \simeq \partial \ln N_g/\partial \ln L$ (Figs. S7G–

H).

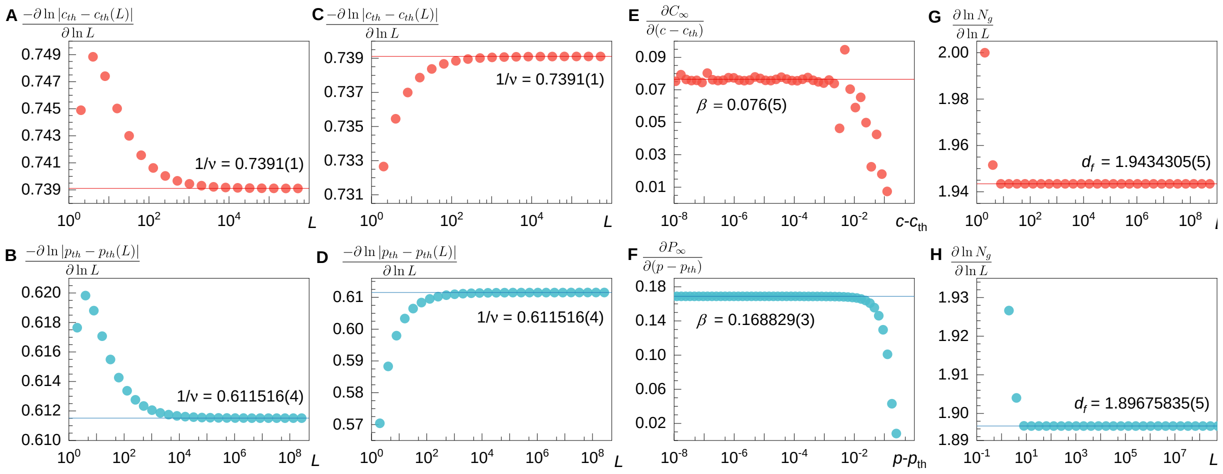

Figure S7: **Numerical calculations of critical exponents.** (**A–D**) Numerical determination of ν. (**A**) quantum $c_{\text{th}}(L) < c_{\text{th}}$ (red) when $n \longrightarrow \infty$, compared to (**B**) classical $p_{\text{th}}(L) < p_{\text{th}}$ (blue). Similarly, (**C**) quantum $c_{\text{th}}(L) > c_{\text{th}}$ (red), compared to (**D**) classical $p_{\text{th}}(L) > p_{\text{th}}$ (blue). (**E–F**) Numerical determination of β for (**E**) concurrence percolation (red) and (**F**) classical percolation (blue). (**G–H**) Using the data from Figs. 3(I) and 3(J), we extract the local slope $\partial \ln N_g / \partial \ln L$ in classical percolation and concurrence percolation, respectively, to determine the fractal dimension d_f.

S6 Asymptotic analysis of critical phenomena on (U, V) flowers

In the following, we calculate the critical thresholds p_{th} (c_{th}), percolating strengths P_∞ (C_∞), and critical exponents (ν, β, d_f) in details, with a focus on the asymptotic limit $V \longrightarrow \infty$.

S6.1 Classical percolation

S6.1.1 Critical threshold p_{th}

We solve the fixed point equation of the RG function:

$$\begin{aligned}
\mathcal{R}(p) &= \text{para}\left(\text{seri}\left(\overbrace{p, p, \dots, p}^{U}\right), \text{seri}\left(\overbrace{p, p, \dots, p}^{V}\right)\right) \\
&= 1 - (1 - p^U)(1 - p^V) \\
&= p^U + p^V - p^{U+V} \\
&= p,
\end{aligned} \tag{S18}$$

which produces

$$p_{\text{th}} \simeq 1 - AV^{-1} + O(V^{-2}) \quad \text{as } V \to \infty, \quad A = \ln \frac{U}{U-1} \simeq \frac{1}{U}. \tag{S19}$$

S6.1.2 Critical exponent ν

In order to find the critical exponent ν, we evaluate

$$\frac{\partial \mathcal{R}(p)}{\partial p}\Big|_{p=p_{\text{th}}} = \frac{\partial (p^U + p^V - p^{U+V})}{\partial p}\Big|_{p=p_{\text{th}}}. \tag{S20}$$

Additionally, we have

$$\lim_{V\to\infty} p_{\text{th}}^U \simeq \lim_{V\to\infty} (1 - AV^{-1})^U \simeq 1 - UAV^{-1} \tag{S21}$$

and

$$\lim_{V\to\infty} p_{\text{th}}^V = \lim_{V\to\infty} (1 - AV^{-1})^V = e^{-A} = \frac{U-1}{U}, \tag{S22}$$

which correspond to the sponge-crossing connectivity *at criticality* along the shorter path and the longer path, respectively.

Substituting p_{th}^U and p_{th}^V into Eq. (S20), we have

$$\begin{aligned}
\frac{\partial \mathcal{R}(p)}{\partial p}\Big|_{p_{\text{th}}} &= p^{-1}(Up^U + Vp^V - (U+V)p^{U+V})\Big|_{p=p_{\text{th}}} \\
&= p^{-1}(Up^U(1-p^V) + Vp^V(1-p^U))\Big|_{p=p_{\text{th}}} \\
&\simeq (1-AV^{-1})^{-1}(U(1-UAV^{-1}) + Ve^{-A}(1-(1-UAV^{-1})) - U(1-UAV^{-1})e^{-A}) \\
&\simeq (1+AV^{-1})(U(1-UAV^{-1})(1-e^{-A}) + UAe^{-A}) \\
&= \left(1-e^{-A}+Ae^{-A}\right)U + \left((1-e^{-A}+Ae^{-A})UA - (1-e^{-A})U^2A\right)V^{-1} \\
&\quad - \left((1-e^{-A})U^2A^2\right)V^{-2}.
\end{aligned} \tag{S23}$$

So the critical exponent ν is given by

$$\begin{aligned}
\nu &= \frac{\ln U}{\ln\left(\frac{\partial \mathcal{R}(p)}{\partial p}\Big|_{p=p_{\text{th}}}\right)} \\
&= \frac{\ln U}{\ln U + \ln(1-e^{-A}+Ae^{-A})} + O(V^{-1}) \\
&= \frac{\ln U}{\ln(1+(U-1)\ln\frac{U}{U-1})} + O(V^{-1}).
\end{aligned} \tag{S24}$$

S6.1.3 Percolating strength P_∞

The non-cluster-defined P_∞ is solved by:

$$\begin{cases}
\begin{cases}
\text{seri}(x',t') = \text{seri}(t, \text{para}(\text{seri}(x, \overbrace{p,p,\ldots,p}^{a}), \text{seri}(y, \overbrace{p,p,\ldots,p}^{U+V-1-a}))) \\
\text{seri}(y',t') = \text{seri}(t, \text{para}(\text{seri}(x, \overbrace{p,p,\ldots,p}^{U-1-a}), \text{seri}(y, \overbrace{p,p,\ldots,p}^{V+a}))) \\
\text{seri}(x',y') = \text{para}(\text{seri}(\overbrace{p,p,\ldots,p}^{U}), \text{seri}(\overbrace{p,p,\ldots,p}^{V}))
\end{cases} & \text{, where } a = 0,1,2,\ldots,U-1; \\
\begin{cases}
\text{seri}(x',t') = \text{seri}(t, \text{para}(\text{seri}(x, \overbrace{p,p,\ldots,p}^{b}), \text{seri}(y, \overbrace{p,p,\ldots,p}^{U+V-1-b}))) \\
\text{seri}(y',t') = \text{seri}(t, \text{para}(\text{seri}(x, \overbrace{p,p,\ldots,p}^{V-1-b}), \text{seri}(y, \overbrace{p,p,\ldots,p}^{U+b}))) \\
\text{seri}(x',y') = \text{para}(\text{seri}(\overbrace{p,p,\ldots,p}^{U}), \text{seri}(\overbrace{p,p,\ldots,p}^{V}))
\end{cases} & \text{, where } b = 0,1,2,\ldots,V-1.
\end{cases} \tag{S25}$$

For the classical case, we know that $\text{seri}(p_1, p_2) = p_1 p_2$ and $\text{para}(p_1, p_2) = 1 - (1 - p_1)(1 - p_2) = p_1 + p_2 - p_1 p_2$. Thus, Eq. (S25) reduces to

$$\begin{cases} \begin{cases} x't' = t(xp^a + yp^{U+V-1-a} - xyp^{U+V-1}) \\ y't' = t(xp^{U-1-a} + yp^{V+a} - xyp^{U+V-1}) \\ x'y' = p^U + p^V - p^{U+V} \end{cases} & \text{, where } a = 0, 1, 2, \ldots, U-1; \\ \\ \begin{cases} x't' = t(xp^b + yp^{U+V-1-b} - xyp^{U+V-1}) \\ y't' = t(xp^{V-1-b} + yp^{U+b} - xyp^{U+V-1}) \\ x'y' = p^U + p^V - p^{U+V} \end{cases} & \text{, where } b = 0, 1, 2, \ldots, V-1. \end{cases} \tag{S26}$$

When $n \to \infty$, we assume $x' = x = y' = y$, thus $xy = \text{para}(p^U, p^V) \overset{p \to p_{\text{th}}}{=} p$ gives $x = y = \sqrt{p}$. By Eq. (S26), the average value satisfies

$$\begin{aligned} \frac{t'}{t} = & \frac{\sum_{a=0}^{U-1} \sqrt{(p^{1/2}p^a + p^{U-1/2}p^V p^{-a} - p^U p^V)(p^{U-1/2}p^{-a} + p^{1/2}p^V p^a - p^U p^V)}}{(U+V)\sqrt{p^U + p^V - p^{U+V}}} \\ & + \frac{\sum_{b=0}^{V-1} \sqrt{(p^{1/2}p^b + p^{U-1/2}p^V p^{-b} - p^U p^V)(p^{V-1/2}p^{-b} + p^{1/2}p^U p^b - p^U p^V)}}{(U+V)\sqrt{p^U + p^V - p^{U+V}}}. \end{aligned} \tag{S27}$$

We denote

$$\begin{aligned} f_1 = & \sum_{a=0}^{U-1} \sqrt{(p^{1/2}p^a + p^{U-1/2}p^V p^{-a} - p^U p^V)(p^{U-1/2}p^{-a} + p^{1/2}p^V p^a - p^U p^V)} \\ \overset{V \to \infty}{\simeq} & \sum_{a=0}^{U-1} \sqrt{(1 + e^{-A} - e^{-A})(1 + e^{-A} - e^{-A})} \\ \overset{V \to \infty}{\simeq} & \; U + O(1), \end{aligned} \tag{S28}$$

and

$$
\begin{aligned}
f_2 &= \sum_{b=0}^{V-1} \sqrt{(p^{1/2}p^b + p^{U-1/2}p^V p^{-b} - p^U p^V)(p^{V-1/2}p^{-b} + p^{1/2}p^U p^b - p^U p^V)} \\
&\overset{V\to\infty}{\simeq} \int_0^V \sqrt{(p^b + p^{U+V-1-b} - p^{U+V-1})(p^{V-1-b} + p^{U+b} - p^{U+V-1})}\, db \\
&= \int_{p^0}^{p^V} \frac{\sqrt{(p^b + p^{U+V-1-b} - p^{U+V-1})(p^{V-1-b} + p^{U+b} - p^{U+V-1})}}{p^b \ln b}\, d(p^b) \\
&\overset{V\to\infty}{\simeq} \int_1^{e^{-A}} -\frac{V}{A} x \sqrt{(x + p^{U+V-1}x^{-1} - p^{U+V-1})(p^{V-1}x^{-1} + p^U x - p^{U+V-1})}\, dx \\
&\overset{V\to\infty}{\simeq} \int_1^{e^{-A}} -\frac{V}{A}(1 + e^{-A}x^{-2} - e^{-A}x^{-1})\, dx \\
&= -\frac{V}{A}(2e^{-A} + Ae^{-A} - 2).
\end{aligned}
\tag{S29}
$$

Therefore,

$$
\frac{P'_\infty}{P_\infty} = \frac{t'}{t} = \frac{f_1 + f_2}{(U+V)\sqrt{p^U + p^V - p^{U+V}}}. \tag{S30}
$$

S6.1.4 Critical exponents d_f and β

Let $\frac{P'_\infty}{P_\infty} = (U{+}V)^{-\theta}$, where the exponent θ traditionally characterizes the size of the giant component at the critical threshold (*24*). When $V \longrightarrow \infty$, we have

$$
\begin{aligned}
V^{-\theta} &\simeq -\frac{V}{A}\frac{2e^{-A} + Ae^{-A} - 2}{(U+V)(1 + O(V^{-1}))} \\
&\simeq -\frac{\ln \frac{U}{U-1}\frac{U-1}{U} - \frac{2}{U}}{\ln \frac{U}{U-1}} + O(V^{-1}) \\
&= -\frac{U-1}{U} + \frac{2}{U}\left(\ln \frac{U}{U-1}\right)^{-1},
\end{aligned}
\tag{S31}
$$

producing

$$
\theta \simeq \frac{\ln\left(\frac{U}{(1-U)+2(\ln\frac{U}{U-1})^{-1}}\right)}{\ln V}. \tag{S32}
$$

The exponent θ is directly related to d_f by $d_f \equiv d(1-\theta)$, therefore,

$$
\begin{aligned}
d_f &\simeq \frac{\ln V}{\ln U}\left(1 - \frac{\ln\left(\frac{U}{(1-U)+2(\ln\frac{U}{U-1})^{-1}}\right)}{\ln V}\right) \\
&\simeq \frac{\ln V}{\ln U} - \frac{\ln\left(\frac{U}{(1-U)+2(\ln\frac{U}{U-1})^{-1}}\right)}{\ln U} + O(V^{-1}).
\end{aligned}
\tag{S33}
$$

Assuming the hyperscaling relation $\beta = \nu(d - d_f)$, we derive

$$\begin{aligned}\beta &\simeq \left(\frac{\ln U}{\ln(1+(U-1)\ln\frac{U}{U-1})} + O(V^{-1})\right)\left(\frac{\ln\left(\frac{U}{(1-U)+2(\ln\frac{U}{U-1})^{-1}}\right)}{\ln U} + O(V^{-1})\right)\\ &\simeq \frac{\ln\left(\frac{U}{(1-U)+2(\ln\frac{U}{U-1})^{-1}}\right)}{\ln(1+(U-1)\ln\frac{U}{U-1})} + O(V^{-1}).\end{aligned} \tag{S34}$$

S6.2 Concurrence percolation

S6.2.1 Critical threshold c_{th}

Let $c = 1 - \frac{m(U,V)V^{-1}}{2}$, thus $c^2 = \left(1 - \frac{m(U,V)V^{-1}}{2}\right)^2 = 1 - mV^{-1} + O(V^{-2})$. We know $\lim_{V\to\infty} c^{2V} = \lim_{V\to\infty}\left(1 - mV^{-1}\right)^V = e^{-m}$ and $c^{2U} \simeq (1 - mV^{-1})^U \simeq 1 - mUV^{-1}$. Thus, the quantum threshold c_{th} is solved by

$$\begin{aligned}\frac{1+\sqrt{1-c^2}}{2} &= \frac{1+\sqrt{1-c^{2U}}}{2}\frac{1+\sqrt{1-c^{2V}}}{2} \Rightarrow\\ \frac{1+\sqrt{1-(1-\frac{m}{V})}}{2} &= \frac{1+\sqrt{1-(1-\frac{m}{V})^U}}{2}\frac{1+\sqrt{1-(1-\frac{m}{V})^V}}{2} \Rightarrow\\ \frac{1}{2}(1+\sqrt{\frac{m}{V}}) &= \frac{1}{4}(1+\sqrt{\frac{mU}{V}})(1+\sqrt{1-e^{-m}}),\end{aligned} \tag{S35}$$

which becomes

$$\frac{1+\sqrt{\frac{mU}{V}}}{2}\cdots\frac{1+\sqrt{1-e^{-m}}}{2} = \frac{1+\sqrt{\frac{m}{V}}}{2} \quad \text{for } V \gg 1. \tag{S36}$$

Noting that $1+\sqrt{1-e^{-m}} \approx 2 - \frac{1}{2}e^{-m}$, multiplying by 2, taking the logarithm and rearranging yields

$$\ln\left(1+\sqrt{\frac{m}{V}}\right) = \ln\left(1+\sqrt{\frac{mU}{V}}\right) + \ln\left(1 - \frac{1}{4}e^{-m}\right). \tag{S37}$$

Using $\ln(1 \pm x) \approx \pm x$ for $x \to 0$, we find

$$\sqrt{\frac{m}{V}} = \sqrt{\frac{mU}{V}} - \frac{1}{4}e^{-m} \quad \text{for } V \gg 1 \tag{S38}$$

or

$$\sqrt{m} = \sqrt{mU} - \frac{1}{4}e^{\frac{1}{2}\ln V - m} \quad \text{for } V \gg 1, \tag{S39}$$

which, divided by $\sqrt{m}$, gives rise to

$$1 = \sqrt{U} - \frac{1}{4}\sqrt{\frac{V}{m}}e^{-m}, \tag{S40}$$

that is,

$$e^{\frac{1}{2}\ln V - m - \frac{1}{2}\ln m} = 4\left(\sqrt{U} - 1\right). \tag{S41}$$

So taking the (natural) logarithm and rearranging, we have

$$m + \frac{1}{2}\ln m - \frac{1}{2}\ln V + \ln\left(4\left(\sqrt{U} - 1\right)\right) = 0 \quad \text{for } V \gg 1. \tag{S42}$$

Hence, we can find m numerically for fixed U as a function of V by solving this equation or Eq. (S39). Note that V only appears via $\ln V$. Hence, there are no numerical problems with solving this equation even for $V \longrightarrow \infty$. Additionally, from Eq. (S42) we have

$$\lim_{V\to\infty} c_{\text{th}}^{U} \simeq 1 - \frac{1}{2}mUV^{-1} \simeq 1 - \frac{1}{4}UV^{-1}\ln V \tag{S43}$$

and

$$\lim_{V\to\infty} c_{\text{th}}^{V} \simeq e^{-m/2} \simeq V^{-\frac{1}{4}}, \tag{S44}$$

which correspond to the sponge-crossing connectivity *at criticality* along the shorter path and the longer path, respectively.

S6.2.2 Critical exponent ν

We denote $\Lambda = \frac{\partial c'}{\partial c}$. When $V \to \infty$, $c' = c$, we have $\Lambda = \frac{\partial c'}{\partial c'^2}\frac{\partial c'^2}{\partial c^2}\frac{\partial c^2}{\partial c} = \frac{1}{2c'}\frac{\partial c'^2}{\partial c^2}2c = \frac{\partial c'^2}{\partial c^2}$, where we write $\frac{\partial C'}{\partial C} = \frac{\partial c'^2}{\partial c^2}$. Using $\frac{1+\sqrt{1-c'^2}}{2} = \frac{1+\sqrt{1-c^{2U}}}{2}\frac{1+\sqrt{1-c^{2V}}}{2}$, we have

$$\begin{aligned}
\frac{\partial\left(\frac{1+\sqrt{1-c'^2}}{2}\right)}{\partial c^2} &= \frac{\partial\left(\frac{1+\sqrt{1-c^{2U}}}{2}\frac{1+\sqrt{1-c^{2V}}}{2}\right)}{\partial c^2} \Leftrightarrow \\
\frac{\partial\left(\frac{1+\sqrt{1-C'}}{2}\right)}{\partial C} &= \frac{\partial\left(\frac{1+\sqrt{1-C^{U}}}{2}\frac{1+\sqrt{1-C^{V}}}{2}\right)}{\partial C} \Leftrightarrow \\
-\frac{1}{\sqrt{1-C'}}\frac{\partial C'}{\partial C} &= \frac{-UC^{U-1}\left(1+\sqrt{1-C^{V}}\right)}{2\sqrt{1-C^{U}}} + \frac{-VC^{V-1}\left(1+\sqrt{1-C^{U}}\right)}{2\sqrt{1-C^{V}}} \Leftrightarrow \\
\frac{\partial C'}{\partial C} &= \frac{C^{-1}\sqrt{1-C'}}{2}\left(\frac{UC^{U}\left(1+\sqrt{1-C^{V}}\right)}{\sqrt{1-C^{U}}} + \frac{VC^{V}\left(1+\sqrt{1-C^{U}}\right)}{\sqrt{1-C^{V}}}\right).
\end{aligned} \tag{S45}$$

When $V \to \infty$, $\lim_{V\to\infty} C^V = \lim_{V\to\infty}(1 - mV^{-1})^V = e^{-m}$, $C^U \simeq (1 - mV^{-1})^U \simeq 1 - mUV^{-1}$, so

$$\Lambda = \frac{1}{2}\left(1 + \frac{m}{V}\right)\sqrt{\frac{m}{V}}\left(U\left(1 - \frac{mU}{V}\right)\frac{1 + \sqrt{1 - e^{-m}}}{\sqrt{1 - \left(1 - \frac{mU}{V}\right)}} + Ve^{-m}\frac{1 + \sqrt{1 - \left(1 - \frac{mU}{V}\right)}}{\sqrt{1 - e^{-m}}}\right). \tag{S46}$$

For large V, approximations are required. Using $\sqrt{1 - x} \approx 1 - \frac{1}{2}x$ for $x \ll 1$, we have $\sqrt{1 - e^{-m}} \approx 1 - \frac{1}{2}e^{-m}$, so

$$\begin{aligned}
\Lambda &= \frac{1}{2}\left(1 + \frac{m}{V}\right)\sqrt{\frac{m}{V}}\left(U\left(1 - \frac{mU}{V}\right)\frac{1 + \sqrt{1 - e^{-m}}}{\sqrt{1 - \left(1 - \frac{mU}{V}\right)}} + Ve^{-m}\frac{1 + \sqrt{1 - \left(1 - \frac{mU}{V}\right)}}{\sqrt{1 - e^{-m}}}\right) \\
&= \frac{1}{2}\left(1 + \frac{m}{V}\right)\sqrt{\frac{m}{V}}\left(U\left(1 - \frac{mU}{V}\right)\frac{2 - \frac{1}{2}e^{-m}}{\sqrt{\frac{mU}{V}}} + Ve^{-m}\frac{1 + \sqrt{\frac{mU}{V}}}{1 - \frac{1}{2}e^{-m}}\right) \\
&= \frac{1}{2}\left(1 + \frac{m}{V}\right)\left(U\left(1 - \frac{mU}{V}\right)\frac{2 - \frac{1}{2}e^{-m}}{\sqrt{U}} + \sqrt{mV}e^{-m}\frac{1 + \sqrt{\frac{mU}{V}}}{1 - \frac{1}{2}e^{-m}}\right) \\
&= \frac{1}{2}\left(1 + \frac{m}{V}\right)\left(\sqrt{U}\left(1 - \frac{mU}{V}\right)\left(2 - \frac{1}{2}e^{-m}\right) + \sqrt{m}e^{\frac{1}{2}\ln V - m}\frac{1 + \sqrt{\frac{mU}{V}}}{1 - \frac{1}{2}e^{-m}}\right) \\
&= \frac{1}{2}\cdot 1\left(\sqrt{U}\cdot 1\cdot 2 + \sqrt{m}e^{\frac{1}{2}\ln V - m}\frac{1}{1}\right) \\
&= \sqrt{U} + \frac{1}{2}\sqrt{m}e^{\frac{1}{2}\ln V - m}.
\end{aligned} \tag{S47}$$

Note that m is determined as a function of U and V by solving

$$m + \frac{1}{2}\ln m - \frac{1}{2}\ln V + \ln\left(4\left(\sqrt{U} - 1\right)\right) = 0 \quad \text{for } V \gg 1. \tag{S48}$$

Hence,

$$\frac{1}{2}\ln V - m = \frac{1}{2}\ln m + \ln K, \text{ where } K = 4\left(\sqrt{U} - 1\right), \tag{S49}$$

and thus

$$\Lambda = \sqrt{U} + \frac{1}{2}\sqrt{m}e^{\frac{1}{2}\ln m + \ln K} = \sqrt{U} + \frac{K}{2}m. \tag{S50}$$

From Eq. (S48), we find m is approximated by $\frac{1}{2}\ln V$ in the limit $V \to \infty$. Therefore, for $V \to \infty$, we have

$$\ln\Lambda \simeq \ln\ln V. \tag{S51}$$

So we conclude that

$$\nu = \frac{\ln U}{\ln \ln V}. \tag{S52}$$

S6.2.3 Percolating strength C_∞

The non-cluster-defined C_∞ is solved by:

$$\begin{cases} \begin{cases} \mathrm{seri}(x', t') = \mathrm{seri}(t, \mathrm{para}(\mathrm{seri}(x, \overbrace{c, c, \ldots, c}^{a}), \mathrm{seri}(y, \overbrace{c, c, \ldots, c}^{U+V-1-a}))) \\ \mathrm{seri}(y', t') = \mathrm{seri}(t, \mathrm{para}(\mathrm{seri}(x, \overbrace{c, c, \ldots, c}^{U-1-a}), \mathrm{seri}(y, \overbrace{c, c, \ldots, c}^{V+a}))) \\ \mathrm{seri}(x', y') = \mathrm{para}(\mathrm{seri}(\overbrace{c, c, \ldots, c}^{U}), \mathrm{seri}(\overbrace{c, c, \ldots, c}^{V})) \end{cases} \text{, where } a = 0, 1, 2, \ldots, U-1; \\ \\ \begin{cases} \mathrm{seri}(x', t') = \mathrm{seri}(t, \mathrm{para}(\mathrm{seri}(x, \overbrace{c, c, \ldots, c}^{b}), \mathrm{seri}(y, \overbrace{c, c, \ldots, c}^{U+V-1-b}))) \\ \mathrm{seri}(y', t') = \mathrm{seri}(t, \mathrm{para}(\mathrm{seri}(x, \overbrace{c, c, \ldots, c}^{V-1-b}), \mathrm{seri}(y, \overbrace{c, c, \ldots, c}^{U+b}))) \\ \mathrm{seri}(x', y') = \mathrm{para}(\mathrm{seri}(\overbrace{c, c, \ldots, c}^{U}), \mathrm{seri}(\overbrace{c, c, \ldots, c}^{V})) \end{cases} \text{, where } b = 0, 1, 2, \ldots, V-1. \end{cases} \tag{S53}$$

When $n \longrightarrow \infty$, $x' = y' = x = y = c^{\frac{1}{2}}$, the quantum series-parallel rules are

$$\mathrm{seri}(c_1, c_2) = c_1 c_2, \tag{S54a}$$

$$\mathrm{para}(c_1, c_2) = \left(1 - \left(\frac{1}{2}\left(1 + \sqrt{1 - c_1^2}\right)\left(1 + \sqrt{1 - c_2^2}\right) - 1\right)^2\right)^{\frac{1}{2}}. \tag{S54b}$$

Thus, C_∞ is simplified to

$$\begin{cases} \begin{cases} c^{\frac{1}{2}} t' = t \left(1 - \left(\frac{1}{2}\left(1+\sqrt{1-c^{2(a+\frac{1}{2})}}\right)\left(1+\sqrt{1-c^{2(U+V-\frac{1}{2}-a)}}\right)-1\right)^2\right)^{\frac{1}{2}} \\ c^{\frac{1}{2}} t' = t \left(1 - \left(\frac{1}{2}\left(1+\sqrt{1-c^{2(U-\frac{1}{2}-a)}}\right)\left(1+\sqrt{1-c^{2(V+a+\frac{1}{2})}}\right)-1\right)^2\right)^{\frac{1}{2}} \\ c^{\frac{1}{2}} c^{\frac{1}{2}} = \left(1 - \left(\frac{1}{2}\left(1+\sqrt{1-c^{2U}}\right)\left(1+\sqrt{1-c^{2V}}\right)-1\right)^2\right)^{\frac{1}{2}} \end{cases} \text{, where } a = 0, 1, 2, \ldots, U-1; \\ \begin{cases} c^{\frac{1}{2}} t' = t \left(1 - \left(\frac{1}{2}\left(1+\sqrt{1-c^{2(b+\frac{1}{2})}}\right)\left(1+\sqrt{1-c^{2(U+V-\frac{1}{2}-b)}}\right)-1\right)^2\right)^{\frac{1}{2}} \\ c^{\frac{1}{2}} t' = t \left(1 - \left(\frac{1}{2}\left(1+\sqrt{1-c^{2(V-\frac{1}{2}-b)}}\right)\left(1+\sqrt{1-c^{2(U+b+\frac{1}{2})}}\right)-1\right)^2\right)^{\frac{1}{2}} \\ c^{\frac{1}{2}} c^{\frac{1}{2}} = \left(1 - \left(\frac{1}{2}\left(1+\sqrt{1-c^{2U}}\right)\left(1+\sqrt{1-c^{2V}}\right)-1\right)^2\right)^{\frac{1}{2}} \end{cases} \text{, where } b = 0, 1, 2, \ldots, V-1. \end{cases} \tag{S55}$$

By Eq. (S55), the average value satisfies

$$\begin{aligned} \frac{t'}{t} = & \frac{\sum_{a=0}^{U-1}\left(\left(1 - \left(\frac{1}{2}\left(1+\sqrt{1-c^{2(a+\frac{1}{2})}}\right)\left(1+\sqrt{1-c^{2(U+V-\frac{1}{2}-a)}}\right)-1\right)^2\right)\left(1 - \left(\frac{1}{2}\left(1+\sqrt{1-c^{2(U-\frac{1}{2}-a)}}\right)\left(1+\sqrt{1-c^{2(V+a+\frac{1}{2})}}\right)-1\right)^2\right)\right)^{\frac{1}{4}}}{(U+V)\left(1 - \left(\frac{1}{2}\left(1+\sqrt{1-c^{2U}}\right)\left(1+\sqrt{1-c^{2V}}\right)-1\right)^2\right)^{\frac{1}{4}}} \\ & + \frac{\sum_{b=0}^{V-1}\left(\left(1 - \left(\frac{1}{2}\left(1+\sqrt{1-c^{2(b+\frac{1}{2})}}\right)\left(1+\sqrt{1-c^{2(U+V-\frac{1}{2}-b)}}\right)-1\right)^2\right)\left(1 - \left(\frac{1}{2}\left(1+\sqrt{1-c^{2(V-\frac{1}{2}-b)}}\right)\left(1+\sqrt{1-c^{2(U+b+\frac{1}{2})}}\right)-1\right)^2\right)\right)^{\frac{1}{4}}}{(U+V)\left(1 - \left(\frac{1}{2}\left(1+\sqrt{1-c^{2U}}\right)\left(1+\sqrt{1-c^{2V}}\right)-1\right)^2\right)^{\frac{1}{4}}}. \end{aligned} \tag{S56}$$

We denote

$$\begin{aligned} f_3 &= \sum_{a=0}^{U-1}\left(\left(1 - \left(\frac{1}{2}\left(1+\sqrt{1-c^{2(a+\frac{1}{2})}}\right)\left(1+\sqrt{1-c^{2(U+V-\frac{1}{2}-a)}}\right)-1\right)^2\right)\left(1 - \left(\frac{1}{2}\left(1+\sqrt{1-c^{2(U-\frac{1}{2}-a)}}\right)\left(1+\sqrt{1-c^{2(V+a+\frac{1}{2})}}\right)-1\right)^2\right)\right)^{\frac{1}{4}} \\ &\overset{V\to\infty}{\simeq} \sum_{a=0}^{U-1}\left(\left(1 - \left(\frac{1}{2}\left(1+O(\sqrt{\frac{\ln V}{V}})\right)\left(1+\sqrt{1-e^{-m}}\right)-1\right)^2\right)\left(1 - \left(\frac{1}{2}\left(1+O(\sqrt{\frac{\ln V}{V}})\right)\left(1+\sqrt{1-e^{-m}}\right)-1\right)^2\right)\right)^{\frac{1}{4}} \\ &\overset{V\to\infty}{\simeq} U\left(1 - O\left(\sqrt{\frac{\ln V}{V}}\right) - O\left(\frac{1}{\sqrt{V}}\right)\right)^{\frac{1}{2}} \\ &\simeq U + O\left(\sqrt{\frac{\ln V}{V}}\right), \end{aligned} \tag{S57}$$

and

$$
\begin{aligned}
f_4 &= \sum_{b=0}^{V-1} \left(\left(1 - \left(\frac{1}{2} \left(1 + \sqrt{1 - c^{2(b+\frac{1}{2})}} \right) \left(1 + \sqrt{1 - c^{2(U+V-\frac{1}{2}-b)}} \right) - 1 \right)^2 \right) \left(1 - \left(\frac{1}{2} \left(1 + \sqrt{1 - c^{2(V-\frac{1}{2}-b)}} \right) \left(1 + \sqrt{1 - c^{2(U+b+\frac{1}{2})}} \right) - 1 \right)^2 \right) \right)^{\frac{1}{4}} \\
&\overset{V\to\infty}{\simeq} \int_{(c^2)^{\frac{1}{2}}}^{(c^2)^{V-\frac{1}{2}}} \left(1 - \left(\frac{1}{2} \left(1 + \sqrt{1 - c^{2(b+\frac{1}{2})}} \right) \left(1 + \sqrt{1 - c^{2(U+V-\frac{1}{2}-b)}} \right) - 1 \right)^2 \right)^{\frac{1}{4}} \\
&\left(1 - \left(\frac{1}{2} \left(1 + \sqrt{1 - c^{2(V-\frac{1}{2}-b)}} \right) \left(1 + \sqrt{1 - c^{2(U+b+\frac{1}{2})}} \right) - 1 \right)^2 \right)^{\frac{1}{4}} \frac{d(c^2)^{b+\frac{1}{2}}}{(c^2)^{b+\frac{1}{2}} \ln (c^2)} \\
&\overset{V\to\infty}{\simeq} \int_{(c^2)^{\frac{1}{2}}}^{(c^2)^{V-\frac{1}{2}}} \frac{-V}{m} \frac{1}{x} \left(\left(1 - \left(\frac{1}{2} \left(1 + \sqrt{1 - x} \right) \left(1 + \sqrt{1 - e^{-m} x^{-1}} \right) - 1 \right)^2 \right) \left(1 - \left(\frac{1}{2} \left(1 + \sqrt{1 - e^{-m} x^{-1}} \right) \left(1 + \sqrt{1 - x} \right) - 1 \right)^2 \right) \right)^{\frac{1}{4}} dx \\
&\simeq \int_1^{e^{-m}} \frac{1}{x} \frac{-V}{m} x^{\frac{1}{2}}\, dx \\
&\simeq \frac{2V}{m} \left(1 - e^{-\frac{m}{2}} \right) \\
&\simeq \frac{4V}{\ln V} + O\left(\frac{V \ln\ln V}{(\ln V)^2} \right).
\end{aligned}
\tag{S58}
$$

Therefore,

$$
\begin{aligned}
\frac{C'_\infty}{C_\infty} &= \frac{t'}{t} \\
&= \frac{f_3 + f_4}{(U+V) \left(1 - \left(\frac{1}{2} \left(1 + \sqrt{1 - c^{2U}} \right) \left(1 + \sqrt{1 - c^{2V}} \right) - 1 \right)^2 \right)^{\frac{1}{4}}} \\
&\simeq \frac{\frac{4V}{\ln V} + O\left(\frac{V \ln\ln V}{(\ln V)^2} \right)}{V \left(1 + O\left(\sqrt{\frac{\ln V}{V}} \right) \right)} \\
&\simeq \frac{4}{\ln V} + O\left(\frac{\ln\ln V}{(\ln V)^2} \right).
\end{aligned}
\tag{S59}
$$

S6.2.4 Critical exponents d_f and β

Similar to the classical case,

$$
\begin{aligned}
d_f &\simeq \frac{\ln V}{\ln U} \left(1 - \frac{\ln\ln V - 2\ln 2}{\ln V} + O\left(\frac{\ln\ln V}{(\ln V)^2} \right) \right) \\
&\simeq \frac{\ln V}{\ln U} - \frac{\ln\ln V}{\ln U} + O\left(\frac{\ln\ln V}{\ln V} \right).
\end{aligned}
\tag{S60}
$$

Assuming the hyperscaling relation $\beta = \nu(d - d_f)$, we derive

$$
\begin{aligned}
\beta &\simeq \left(\frac{\ln U}{\ln\ln\sqrt{V}} + O\left(\frac{\ln\ln\ln V}{(\ln\ln V)^2} \right) \right) \left(\frac{\ln\ln V}{\ln U} + O\left(\frac{\ln\ln V}{\ln V} \right) \right) \\
&\simeq 1 + O\left(\frac{\ln\ln\ln V}{\ln\ln V} \right).
\end{aligned}
\tag{S61}
$$

S6.3 Comparison with numerical results

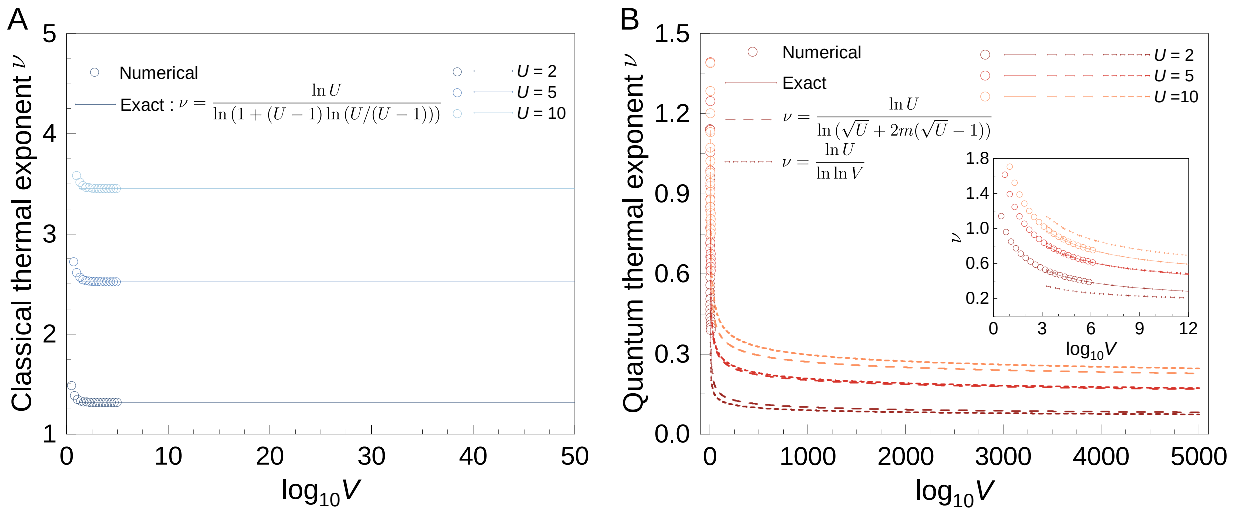

Figure S8: **The critical exponent ν as a function of V given the shortest path $U = 2, 5, 10$.** (**A**) Classical percolation. Open blue circles are simulation values using finite-size analysis. The colors from darker blue to lighter blue are for $U = 2, 5, 10$ respectively. Solid line is derived theoretical solution, which is a constant equal to $\ln U/\ln(1 + \ln U)$ for $V \to \infty$ and is larger when U increases. (**B**) For quantum percolation, when U is fixed, ν exhibits a different asymptotic behavior. The lines from top to bottom demonstrates decreasing degree of approximation [Eqs. (S46), (S50), and (S51)], respectively for Λ in the equation $\nu = \ln U/\ln \Lambda\big|_{c=c_{\rm th}}$. Open red circles are simulation values using finite-size analysis. The solid light red line is derived theoretical solution. The colors from darker orange to lighter orange are for $U = 2, 5, 10$ respectively. The quantum ν decreases as V increases, and will eventually reach $\nu = 0$ for any constant U. Even though $\nu \to 0$ for $V \to \infty$, the non-zero values of ν observed for V finite ($V \approx 10^{5000}$) might be of physical relevance for a QN.

For (U, V) flowers with different U and V, these critical exponents have very different asymptotic behaviors. We generalize above results to the asymptotic limits of constant U and $V \to \infty$ and find that the classical exponent ν does not change. For example, $U = 2$, given the expression of the classical exponent ν, it must be greater than the lower bound $\nu \approx 1.316$ (Fig. S8A) when V increase. By comparison, in the quantum case the exponent ν decreases with V and will eventually

reach the value of 0 for $V \to \infty$ (Fig. S8B). These results demonstrate that the classical exponent ν is independent on V while the quantum exponent ν changes with both U and V. In addition, for larger constant U, the limit value of the classical exponent ν also increases, implying that U plays a decisive role in the classical case. While for the quantum case, the ν for $U = 2, 5, 10$ are closer compared with the classical case. But the distinctions still exist in the speed of going to zero due to the weighting factor related to the shortest path U in quantum percolation.

S7 Analysis on real-world Internet network

We analyzed the effect of longer paths in concurrence percolation compared to classical percolation on a real autonomous-system-level Internet network (Fig. S9). The detouring process required that the path lengths of level k in the network should grow by a factor of q^k. We focused on two distinct levels of path lengths, corresponding to path numbers of $(1, 3)$, respectively; thus the growth multiples were $(1, q)$.

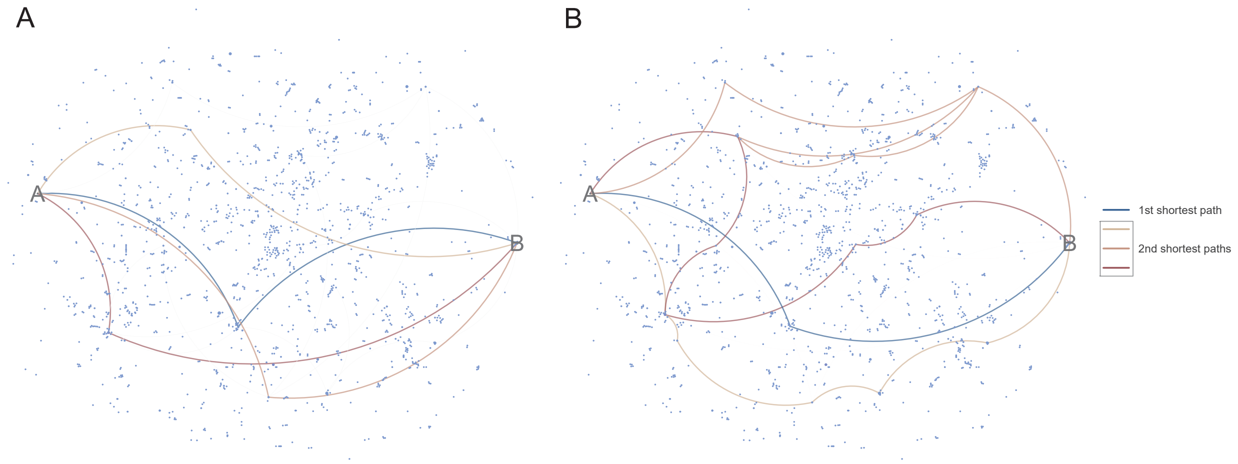

Figure S9: **Illustration of the Internet including a focused subgraph of the Internet, centered around two hubs A and B.** The subgraphs highlight multiple hierarchical and intersecting paths of varying lengths, ranging from the shortest to longer routes. (**A**) The initial subgraph g_1 ($q = 1$). (**B**) The subgraph g_q with q-times longer paths between the same hubs ($q = 3$).

The detouring process goes as follows: First, an initial subgraph g_1 ($q = 1$) was constructed,

composed of four shortest non-overlapping paths between two randomly selected nodes. On this basis, for $q > 1$, the first-level shortest path was kept unchanged, while the three second-level paths were replaced by q-times longer paths between the same nodes, producing a new subgraph g_q. All paths must have no overlaps. During the process, the selection of nodes was flexible, with the only constraint that all the subgraphs with non-overlapping paths were able be found between them. Under this constraint, we selected 10 random pairs of nodes with large enough node degrees, which should be no less than 7. At the same time, since all the paths of g_q were only length-fixed, it was possible to find more than one g_q from the original real network. We randomly selected 20 g_q for each pair of nodes. As a result, for each q, the simulation involved 10 different g_1 and 200 different g_q in total.

We located the finite-size percolation thresholds of all g_q under different path lengths at $P_{\mathrm{sc}} = 0.99$ (in classical case) or $C_{\mathrm{sc}} = 0.99$ (in quantum case), where $q = 2, 3, \ldots, 8$. For each q, the thresholds were averaged and substituted into $A_x(q)$ [Eq. (7) in the main text], yielding the final results (Fig. 4C in the main text).
